# Zinc Oxide Nanoparticles Affect the Genomic and Redox Status of Chicken Embryo—Influence of Shape

**DOI:** 10.3390/nano15181412

**Published:** 2025-09-13

**Authors:** Bartłomiej Dominiak, Julita Rosowska, Agnieszka Wal, Alicja Majewska, Bartłomiej S. Witkowski, Łukasz Wachnicki, Jarosław Kaszewski, Anna Słońska, Joanna Cymerys, Mikołaj A. Gralak, Marek Godlewski, Michał M. Godlewski

**Affiliations:** 1 Department of Physiological Sciences, Institute of Veterinary Medicine, Warsaw University of Life Sciences—SGGW, Nowoursynowska 159, 02-776 Warsaw, Poland; bartlomiej_dominiak@sggw.edu.pl (B.D.); julita_rosowska@sggw.edu.pl (J.R.); alicja_majewska@sggw.edu.pl (A.M.); gralakmikolaj@yahoo.co.uk (M.A.G.); michal_godlewski@sggw.edu.pl (M.M.G.); 2 Institute of Physics PAS, Polish Academy of Sciences, Al. Lotników 32/46, 02-668 Warsaw, Poland; bwitkow@ifpan.edu.pl (B.S.W.); lwachn@ifpan.edu.pl (Ł.W.); kaszewski@ifpan.edu.pl (J.K.); 3 Department of Plant Physiology, Institute of Biology, Warsaw University of Life Sciences—SGGW, 02-776 Warsaw, Poland; agnieszka_wal@sggw.edu.pl; 4 Division of Microbiology, Department of Preclinical Sciences, Institute of Veterinary Medicine, Warsaw University of Life Sciences—SGGW, Ciszewskiego 8, 02-786 Warsaw, Poland; anna_slonska-zielonka@sggw.edu.pl (A.S.); joanna_cymerys@sggw.edu.pl (J.C.)

**Keywords:** ZnO nanoparticles, shape of the nanoparticles, embryotoxicity, full genomic microarrays, oxidative stress, chicken embryo model

## Abstract

With the spread of nanotechnology use in industry, exposure to nanomaterials is currently exponentially increasing. With reports indicating nanoparticles’ ability to pass through key biological barriers—gastrointestinal, lung, skin, blood-brain and the placenta barriers—the question of their safety, particularly the risks associated with embryonic development, arises. The aim of this article is to verify the impact of ZnO nanoparticles, which are commonly used and considered to be safe for adult organisms on the developing embryo. In the current study, the influence of the dose and shape of ZnO nanoparticles (oval vs. long) was evaluated in the chicken embryo model. The oxidative stress (superoxide dismutase (SOD)) activity, malondialdehyde (MDA) and carbonylated protein ((CP) levels), and gene expression changes (full genomic microarray study) were tested. We found that at both doses (10 µg/mL and 100 µg/mL, 100 µL into the air chamber) neither elongated nor oval ZnO nanoparticles changed in ovo mortality. Long ZnO nanoparticles had a lesser and more delayed impact on evaluated parameters, regardless of their higher in vitro toxicity. However, both nanoparticle forms induced changes in the oxidoreductive potential and affected expression of a significant number (1487 for oval and 548 for long ZnO nanoparticles) of identified genes during early embryo development.

## 1. Introduction

Nanoparticles (NPs) are materials that in at least one dimension have a size in the range of 1 nm to 100 nm [1]. Over the last few years, we have seen an increase in the popularity of nanomaterials, as well as the number of products containing them. So far, NPs have found applications as food additives or in food packaging, and in the cosmetics industry, in body products including sunscreen creams or toothpaste. They are also used in the clothing and electronics industries, as well as in dentistry as dental components [2]. With the widespread use of NPs and the increase in human exposure to them, the question of safety is becoming increasingly important. NPs are capable of penetrating biological barriers such as the gastrointestinal, lung, skin, blood–brain barrier and the placenta [3,4]. Therefore, when discussing the use of NPs and considering intentional and unintentional exposure of the humans or animals, it is important to carry out extended toxicological studies, including verification of potential embryotoxicity. In addition, due to the numerous modifications that NPs can undergo and the different production methods that often confer different physical–chemical properties, NPs can alter their behaviour under biological conditions, which in turn may result in different toxicological results [5].

Some of the most popular NPs that are used in industry and in everyday products are ZnO NPs [2,6]. Their solubility in biological media is an essential characteristic from the point of view of toxicology. This results in a simultaneous effect of the NPs themselves and the Zn^2+^ ions released [7,8,9]. One of the most harmful effects of metal oxide nanoparticles is related to oxidative stress, which is a result of reactive oxygen species (ROS) generation, which in the case of ZnO NPs is the effect of both NPs and released ions [7]. This can result in both direct cytotoxicity and changes at the genetic level [10], which can be particularly harmful in the case of embryonic development [7,11,12].

The toxicological assessment of nanoparticles has been extensively discussed in the scientific literature. However, variations in experimental models, administered doses, nanoparticle provenance, and manufacturing techniques—which frequently result in distinct physicochemical characteristics—complicate the integration and direct comparison of the available datasets.

In a zebrafish embryo model [13], exposure to ZnO nanoparticles (10–30 nm, no shape information) at concentrations of 1, 5, 10, 20, 50, and 100 ppm for 96 h after fertilisation caused toxicity to the developing nervous and vascular systems, with effects occurring at much higher levels than in exposure to pure Zn^2+^ ions. Interestingly, abnormal secondary motoneuron axon phenotypes were also observed in generations F1 and F2, indicating long-term ZnO nanoparticle toxicity. Yan et al. [7] tested the effect of ZnO nanoparticles on cranial neural crest cells (CNCCs) during embryogenesis of a chicken embryo. In their study, 100 μL of ZnO nanoparticles at 50 μg/mL was applied into the air chamber of chicken embryos from incubation day zero to nine or twelve. Embryos exposed for nine and twelve days showed varying degrees of deformation in the craniofacial skeleton. Both ZnO nanoparticles and released Zn^2+^ ions produced ROS that contributed to cellular toxicity, inflammation, and apoptosis, mediated by NF-κB signalling cascades and mitochondrial dysfunction. These events suppressed production and migration of CNCCs, leading to craniofacial malformations. For embryos up to 10 on the Hamburger–Hamilton scale (10 HH, 33–38 h post-fertilisation), an increase in mortality and shortened embryo length was observed. Again, it must be noted that these negative effects were observed at a combined dose that far exceeded natural exposure. In another avian study, Hao and colleagues [14] fed hens with ZnO nanoparticles at 200 mg/kg of feed for 24 weeks. After artificial insemination, liver samples from F1 chickens were collected at embryonic day 18, and at 3, 5, 10, and 20 days post-hatching. Genes associated with lipid synthesis and liver growth were downregulated in controls, whereas genes related to mitochondrial and oxidative stress were upregulated in the ZnO-exposed group, along with increased apoptosis markers, evidence of cell damage, and pathological changes. The authors suggested a potential embryotoxic effect, but did not quantify the nanoparticle amount that directly interacted with embryos. Furthermore, the combined dose greatly exceeded EU regulatory limits [15]. In the mouse model study [16], 30 nm ZnO nanoparticles were administered orally to pregnant females at 20, 60, 180, or 540 mg/kg body weight from gestation days 10.5 to 17.5. No change in maternal weight was seen at the lowest doses, whereas at higher concentrations, maternal body weight was reduced. Significant decreases in foetal weight and malformations were observed only at 540 mg/kg. Pathological evaluation showed decreased and structurally damaged placental spongiotrophoblast areas, likely resulting from placental oxidative stress, endoplasmic reticulum stress, and apoptosis. Importantly, negative effects in this model were also only observed at doses that extremely exceeded possible natural exposure.

ROS are molecules formed as a result of incomplete reduction or excitation of oxygen (O_2_). They include, among others, the superoxide anion radical (O_2_•^−^), hydroxyl radical (•OH), and hydrogen peroxide (H_2_O_2_) [17]. The biological function of ROS depends on their concentration within the cell. At low levels, they act as signalling molecules, whereas their excessive accumulation can potentially lead to cell deterioration and cell death [18]. In animal cells, the neutralisation of ROS is primarily carried out by an integrated antioxidant defence system consisting of enzymatic components such as superoxide dismutase (SOD) and catalase (CAT), as well as non-enzymatic antioxidants. SOD plays a particularly crucial role as the first line of defence, catalysing the dismutation of the O_2_•^−^ into H_2_O_2_ and O_2_ [19]. Increased SOD expression under stress conditions serves as an adaptive mechanism to maintain redox homeostasis and prevent oxidative damage [20]. ROS react with cellular components such as proteins, lipids, and nucleic acids [21]. They modify amino acid residues in proteins, resulting in post-translational modifications (PTMs), such as carbonylation. Protein carbonylation is one of the most common and irreversible oxidative modifications. Carbonyl groups introduced into arginine (Arg), proline, threonine, or lysine residues alter the biological activity of proteins, promoting their aggregation [22]. As these oxidised protein aggregates are poorly degraded by cellular systems, their persistence can contribute to cellular dysfunction and has been observed in various physiological disorders and diseases, e.g., Alzhimer’s [23]. Due to the chemical stability of the carbonyl groups, the content of proteins with these PTMs is considered a reliable and widely used indicator of protein oxidation in biological research, also referred to as a biomarker of oxidative stress [24].

ROS react with lipids present in cell membranes, leading to their peroxidation. As a result of this reaction, lipid hydroperoxides and various aldehydes are formed, including malondialdehyde (MDA) and propionaldehyde. These molecules can act as signalling compounds, activating cellular protective (antioxidant) mechanisms. However, their excessive accumulation leads to autophagy, apoptosis, or cell death [25,26]. Among lipid peroxidation-derived aldehydes, MDA is considered a reliable and commonly used indicator of oxidative stress, primarily due to its chemical stability and capacity to diffuse across membranes.

Therefore, the aim of the current article is to test the effect of the dose and shape of ZnO NPs obtained by the hydrothermal method on oxidative stress and to evaluate their impact level of changes at the genomic level, using an in ovo chicken embryo model.

## 2. Materials and Methods

### 2.1. Nanoparticle Synthesis

Two types of ZnO NPs were synthesised using the microwave hydrothermal method: undoped long ZnO (ZnO LONG) and oval ZnO doped with 0.5% mol of europium (ZnO OVAL). Distinct microwave hydrothermal processes were employed, utilising various chemical reagents and conducted at different pressures (Table 1). Synthesis of the samples involved two main stages: the preparation of the initial solution and alkalisation, followed by hydrothermal treatment in a reactor [27]. At the beginning of each synthesis, appropriate amounts of inorganic salts were accurately weighed and dissolved in 200 mL of distilled water at room temperature (RT). Once a clear solution was achieved, the alkalisation process was initiated by the gradual, dropwise addition of a precipitating agent under continuous stirring with a magnetic stirrer, until the desired pH of the solution was reached.

The resulting precipitate was then filtered and thoroughly washed with distilled water. After washing, it was transferred into a Teflon vessel, which was filled with distilled water up to 80% of its volume, in preparation for the microwave hydrothermal treatment. The hydrothermal reaction, assisted with microwaves, was carried out in a Magnum II reactor (Ertec, Poland) equipped with 700 W microwave heating. Operational parameters were controlled using Magnum V2 software (version 1.9.8.14). For the undoped ZnO LONG, the process was conducted under a pressure of 6 MPa, while the Eu-doped ZnO OVAL NPs were subjected to a pressure of 8 MPa. The maximum temperature was set at 300 °C, and the hydrothermal process lasted for 20 min. After reaction, the products were transferred to a crystalliser and dried at 60 °C for 24 h. Final step involved mechanical grinding of the dried product using an agate mortar.

### 2.2. Experimental Methods for Nanoparticle Characterisation

The crystalline phase of the NPs was determined using X-ray diffraction (XRD) analysis conducted on a Panalytical X’Pert Powder Diffractometer (Westborough, MA, USA) with CuKα radiation (λ = 1.54060 Å). Data were collected in the range of 20° ≤ 2θ ≤ 70° at RT, using a step size of 0.05°. The surface morphology and size distribution of the ZnO NPs were examined through scanning electron microscopy (SEM), utilising a Hitachi SU-70 (Hitachi High-Tech Corporation, Tokyo, Japan), with a resolution step of 1 nm. This device was equipped with an energy dispersive X-ray (EDX) detector for elemental composition analysis, as well as a GATAN Mono CL3 system for cathodoluminescence (CL) measurements. Additionally, the photoluminescence emission (PL) and excitation (PLE) spectra of the ZnO nanoparticles were measured at RT using a Horiba/Jobin-Yvon Fluorolog-3 spectrofluorometer. The setup included a 450 W xenon lamp as the excitation source, an iHR550 monochromator for wavelength selection, and a Hamamatsu R928P photomultiplier for detection. The electrostatic stability and dispersity of ZnO nanoparticles in suspension were examined by assessing their zeta potential and hydrodynamic diameter. Measurements were performed at ambient conditions (~25 °C) using a Zetasizer Ultra system (Malvern Panalytical, Malvern, UK), equipped with modules for dynamic light scattering (DLS) and electrophoretic light scattering (ELS). Nanoparticles were initially dispersed in deionised water, followed by ultrasonic processing using Sonic VCCX500 system (Sonic and Materials Inc., Newtown, CT, USA) operated for 4 min, to minimise agglomerate formation. All measurements were performed without pH modification. Each sample was measured five times, and the results were expressed as average values with standard deviations.

### 2.3. Isolation of Chicken Embryo Fibroblasts (CEFs)

Primary chicken embryo fibroblasts (CEFs) were isolated from 12-day-old chicken embryos, following the protocol described by Hernandez and Brown [28]. Briefly, embryos were aseptically removed and dissected to eliminate non-fibroblast tissues. The remaining tissue was macerated, washed with phosphate-buffered saline (PBS) containing calcium and magnesium, and digested with 0.25% trypsin-EDTA (Gibco Life Technologies, Paisley, UK) at 37 °C for 15–20 min. The resulting cell suspension was filtered, centrifuged (5 min, 1200 rpm), and resuspended in Dulbecco’s Modified Eagle Medium (DMEM) with GlutaMAX^TM^ supplemented with 10% foetal bovine serum (FBS) and 1% penicillin/streptomycin (Gibco Life Technologies). Cells were counted and seeded at a density of 1 × 10^6^ cells per 25 cm^2^ flask and cultured in a humidified incubator at 37 °C with 5% CO_2_.

### 2.4. Cell Viability Measurement

CEFs were seeded at a density of 1 × 10^6^ cells/well in 24-well cell culture plates and incubated under standard conditions until they had 80–90% confluence. Cells were treated with decreasing concentrations (from 1 to 0.001 mg/mL) of NPs for 24 h. The cell viability of CEFs was examined by trypan blue stain (Sigma-Aldrich). Following treatment, cells were harvested by digestion with 0.2% trypsin-EDTA, resuspended in 1 mL PBS, and mixed with trypan blue (Sigma-Aldrich). Cell viability was determined using an automated cell counter (Olympus Cell Counter, model R1), and results were expressed as the percentage of viable cells. All experiments were performed in quadruplicate.

### 2.5. In Ovo Experimental Model

**Figure 1:**
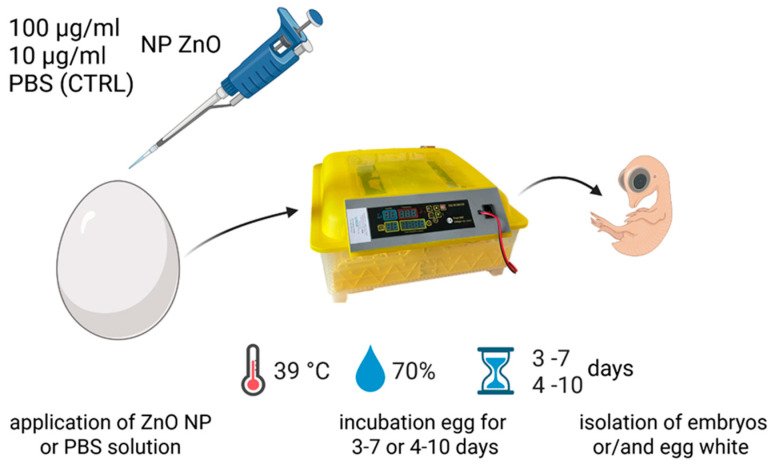
ZnO NPs (for experimental groups) or PBS (for CTRL) were applied to the air chamber before the start of egg incubation. Eggs used for AAS analysis were incubated from days 3 to 7, and embryos collection for SOD activity, MDA and protein carbonylation analysis were collected from days 4 to 10 of incubation. Figure created with the use of Biorender application: https://app.biorender.com/citation/685edab181f997d71c844f97 (accessed on 10 September 2025).

ZnO NPs of both long (ZnO LONG) and oval (ZnO:Eu–ZnO OVAL) shapes were used for biological studies at two concentrations 10 µg/mL and 100 µg/mL in PBS). As an experimental model, the in ovo model was used; after drilling a hole in the shell, the NPs suspensions (100 µL per egg) were administrated to the air chamber [7] of fertilised Ross 308 eggs (Figure 1). The hole was then sealed with parafilm. According to regulations, no local ethical committee agreement was required, as the final embryonic development day at the sample collection did not exceed day 12. The control group (CTRL) received 100 µL of PBS. The incubation process was carried out at a temperature of 39 °C and a humidity of 70% within commercial egg incubators with egg rotation set at hourly intervals. The embryo mortality was assessed at the sample selection day, with no changes observed between NPs treatments and the CTRL.

To verify the distribution of Zn concentration after administration of ZnO nanoparticles using atomic absorption spectrometry (AAS), the collection of embryos and egg white samples was performed at days 3, 4, 5, 6 and 7 of incubation.

Based on the results from the AAS, the measurement of activity of SOD and content of protein carbonyl and MDA as the markers of the oxidative stress was performed for chicken embryos between days 4 and 10 of incubation (Figure 1).

### 2.6. Measurement of Zn Concentration with Flame Atomic Absorption Spectrometry (AAS)

Chicken embryos and egg white samples, isolated at days 3, 4, 5, 6 and 7 of incubation following in ovo application of ZnO LONG or ZnO OVAL NPs (10 and 100 μg/mL) or PBS (CTRL), were weighed and transferred into high-pressure vessels. Each sample was treated with 2 mL of 65% nitric acid and 1 mL of 30% hydrogen peroxide (Merck, Poznań, Poland) and left for 18 h. Mineralisation was then performed using the Ethos 900 microwave digestion system (Milestone, Sorisole, Italy). Zinc concentrations in the digested samples were determined using AAS (PerkinElmer 1100B, Shelton, CT, USA). Experimental data was collected from 7 to 22 eggs for each experimental group per each day, with experiments conducted in 2–3 repetitions.

### 2.7. Confocal Microscopy

To assess the fluorescence of the nanoparticles and to confirm the distribution of ZnO OVAL nanoparticles within the tissues of developing embryos, confocal microscopy was employed. Chicken embryos were collected on days 5, 7, and 10 of incubation. The embryos were fixed in formalin for 24 h. and subsequently transferred to 70% ethanol. For paraffin embedding, the samples were processed through a graded ethanol series (70%, 80%, 96%, 100%), followed by xylene, and finally infiltrated with paraffin using a tissue processor (STP 120, Microm, Walldorf, Germany). The paraffin-embedded blocks were sectioned at a thickness of 5 µm. The prepared sections were then deparaffinised and stained with 7-aminoactinomycin D (7-AAD, Cayman Chemical, MI, USA) to visualise cell nuclei. Imaging was performed at 20x lens under FV-500 Olympus Scanning Confocal System (Olympus Poland). The Following Excitation-emission filters were used: 405 nm vs. 430–450 nm; 488 nm vs. 505–525 nm; 543 nm vs. 560 IF. Confocal microscopy figures were combined in Adobe Photoshop CS6; original microphotographs are attached as a Supplementary Materials to the current article (Figures S1–S12).

### 2.8. Measurement of Superoxide Dismutase (SOD) Activity

SOD activity was measured with the spectrophotometric method [29]. A total of 200 mg of isolated chicken embryo tissues were homogenised in 1 mL of protein isolation buffer (PBS, 1 mM Ditiotreitol (DTT), 1 mM Phenylmethanesulfonyl fluoride (PMSF), 3 mM EDTA, 3 mM MgSO_4_). Homogenates were centrifuged 12,000× *g* for 10 min at 4 °C and the supernatant was collected to the eppendorf tubes. To the 100 µL of protein extract, 200 µL methionine 0.19 M, 200 µL riboflavin 0.11 mM, 100 µL Nitro blue tetrazolium chloride (NBT) 1.7 mM and 1400 µL PBS were added. To the blank, protein isolation buffer was added instead of protein extract. Absorbance was measured at 560 nm. The results were expressed as U/µg protein. A total of 1 U of SOD activity is the amount of extract that inhibits the formation of formazan by 50%. The experiments were performed in 6 biological replicates and in 3 technical repetitions.

### 2.9. Measurement of Protein Carbonyl (CP) Content

CP content was measured with the spectrophotometric method [30]. Proteins were isolated as described above. Proteins were diluted in PBS to a concentration of 1 µg/µL. To 400 µL of protein extract, 10 mM 2,4-dinitrophenylhydrazine (DNPH) in 0.5 M phosphoric acid (H_3_PO_4_) was added, in a 1:1 ratio. The mixtures were incubated for 10 min at RT. In blank, 0.5 M H_3_PO_4_ without DNPH was added. Then, 200 µL 6 M NaOH was added, and all samples were incubated for 10 min at RT. Following this, the absorbance was measured at 450 nm. Concentrations of protein carbonyl were calculated with extinction coefficient ε = 22,308 M^–1^ cm^–1^. The protein carbonyl content was expressed as nmol/µg protein. The experiments were performed in 6 biological replicates and in 3 technical repetitions.

### 2.10. Measurement of Malondialdehyde (MDA) Level

The level of MDA was measured with spectrophotometric method [31]. A total of 200 mg of isolated embryonic tissues were homogenised in 1 mL of 0.1% Trichloroacetic acid (TCA) with 0.01% Butylated hydroxytoluene (BHT). Homogenates were centrifuged 10,000× *g* for 15 min at 4 °C and the supernatant was collected to the eppendorf tube. The supernatant, PBS, and 0.5% thiobarbituric acid (TBA) prepared in 20% TCA were mixed in ratio 1:1:2. To the blank, 0.1% TCA was added instead of supernatant. All samples were incubated for 30 min at 95 °C and cooled before the measurement. Absorbance of cooled samples was measured at 532 nm. Concentrations of MDA were calculated with extinction coefficient ε = 15.5 M^–1^ cm^–1^. Content of MDA was expressed as nmol/g tissue. The experiments were performed in 6 biological replicates and in 3 technical repetitions.

### 2.11. Microarray Study

**Figure 2:**
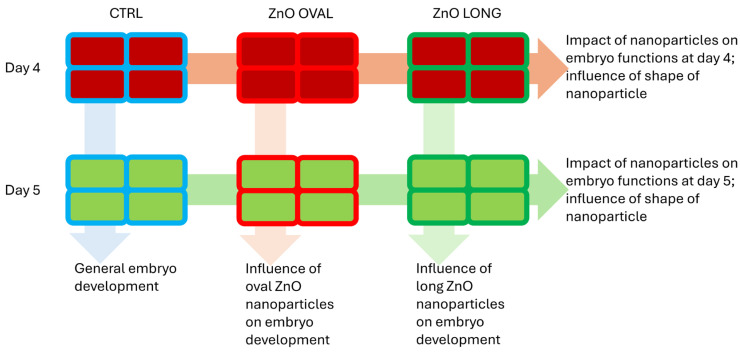
Diagram presenting the schematics of microarray analysis. Results of the microarray study were analysed horizontally (impact of the OVAL and LONG NPs vs. CTRL at days 4 and 5) and vertically showing nanoparticle-specific changes for ZnO OVAL and ZnO LONG in gene expression between days 4 and 5 with exclusion of changes occurring during normal growth of chicken embryo (CTRL progression). Outline colours (blue—CTRL, red—ZnO OVAL, green—ZnO LONG), shape fill colours (red—day 4, green—day 5).

For this part of the experiment, RNA was isolated from chicken embryos incubated for 4 and 5 days, following NPs application (10 µg/mL of ZnO OVAL or ZnO LONG nanoparticles suspended in PBS, 100 µL per egg). The control group was treated with 100 µL of PBS. The schematic design of the microarray experiment is presented on Figure 2. Total RNA was extracted using the RNeasy Mini Kit (Qiagen, Hilden, Germany). During RNA isolation, DNase (RNase-Free DNase Set, Qiagen, Hilden, Germany) was used to purify the isolated RNA from DNA contamination. The entire procedure was performed according to the manufacturer’s protocol. The NanoDrop™ 2000 spectrophotometer (Thermo Fisher Scientific, Waltham, MA, USA) was used to assess the RNA purity and concentration. RNA integrity was verified using the Agilent Bioanalyzer 2100 with the Agilent RNA 6000 Nano Kit (Agilent Technologies, Palo Alto, CA, USA). Only samples revealing high integrity (RIN = 10) were used for microarray. Twenty-four gene expression microarrays were performed to identify genes with altered expression. The experiment utilised chicken (V2) gene expression microarrays, 4x44k (Agilent Technologies, USA) and an Agilent Technologies reagent set, according to the manufacturer’s protocol. Samples for hybridisation were prepared using the low input quick amp labelling kit, one colour to amplify and label (Cy3) target RNA and to generate complementary RNA (cRNA), with the RNA spike-in kit as an internal control, and the gene expression hybridization kit. The microarrays were hybridised at 65 °C for 17 h in the hybridisation oven. After hybridisation, the microarrays were washed using dedicated buffers from the gene expression wash buffer kit (Agilent Technologies, USA). Acquisition and analysis of hybridisation intensities were performed using the Agilent DNA Microarray Scanner G2505C. Agilent Feature Extraction (FE) Software, version 10.7.3.1 was used to extract data from microarrays and subtract the background.

### 2.12. Quantitative PCR

To verify microarray results, the expression of selected genes: *COL6A2* (collagen alpha-2(VI) chain), *SDHA* (succinate dehydrogenase [ubiquinone] flavoprotein subunit, mitochondrial), *PSMB7* (proteasome 20 S Subunit Beta 7), *STEAP3* (STEAP3 metalloreductase) were analysed using real-time PCR. Commercial primer sets were used to amplify all selected genes (PrimePCR SYBR Green Assay, Chicken, Bio-Rad, Warsaw, Poland). RPL13 (60 S ribosomal protein L13) gene was used as a reference gene, after verification by the geNorm algorithm as the most stable gene. Script cDNA Synthesis Kits (Bio-rad) was used for reverse transcription according to the manufacturer’s procedure. The real-time PCR reaction was performed using iTaq Univer SYBR Green Supermix set (Bio-Rad) on termocycler Aria Mx (Agilent Technologies). The conditions of real-time PCR reactions were as follows: polymerase activation at 95 °C for 2 min; amplification (40 cycles) including denaturation at 95 °C for 5 s, and primer annealing and extension at 60 °C for 30 s. The results were calculated using the 2^−ΔΔCt^ method [32].

### 2.13. Statistical Analysis

For statistical evaluation, all acquired results were initially tested for equal standard deviation and Gaussian distribution (Kolmogorov and Smirnov test). According to the results, the following statistical analysis was performed with either ANOVA followed by Tuckey post hoc test for Gaussian distribution, or Mann–Whitney test for non-Gaussian distribution. All statistical analysis was performed in the Graph-Pad In-Stat 3.10 software, with *p* ≤ 0.05 considered significant, *p* ≤ 0.01 or *p* ≤ 0.001 as highly significant, and *p* ≤ 0.1 a trend.

The statistical analysis of microarrays was performed using Gene Spring14 software (Agilent, Santa Clara, CA, USA). The probe sets were filtered by flags to remove poor quality probes (absent flags). The statistical significance of the differences observed was evaluated using a Moderated T-test (*p* < 0.05). Multiple testing correction was performed using the Benjamini and Hochberg false discovery rate (FDR < 0.05). Microarray data were deposited in the Gene Expression Omnibus data repository (accession number: GSE301197).

## 3. Results

### 3.1. Results—Physical Study

#### 3.1.1. Structural Analysis Based on X-Ray Diffraction (XRD) Measurements

**Figure 3:**
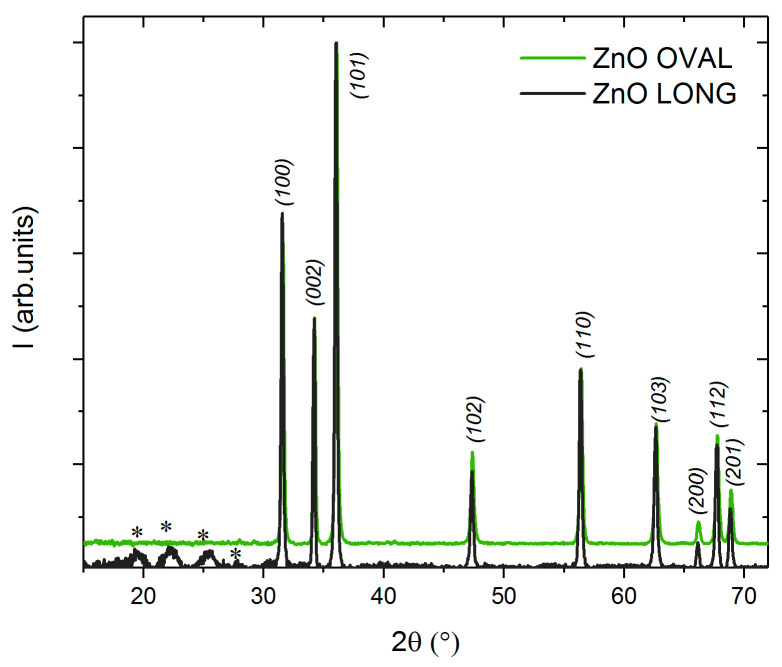
X-ray diffractograms (XRD) of ZnO LONG NPs (black line) obtained from zinc acetate and ZnO OVAL NPs (green line) synthesised from zinc nitrate in a microwave-assisted hydrothermal process. Asterisks indicate reflections originating from zinc hydroxycarbonate complexes, which are by-products of the synthesis.

XRD patterns of both ZnO OVAL and ZnO LONG NPs are shown in Figure 3. The diffractograms exhibit reflections corresponding to the (100), (002), (101), (102), (110), (103), (112), and (201) planes, which are characteristic of hexagonal ZnO (wurtzite, PDF card no. 36-1451). In the case of ZnO LONG synthesised using zinc acetate and ammonium hydroxide solution, additional weak reflections appear in the range of 2θ: 20–30°, indicating the presence of traces of secondary phases. However, the significant broadening of these peaks complicates their reliable identification.

For ZnO OVAL NPs synthesised from zinc nitrate, only very weak additional reflections are observed in the 15° < 2θ < 30° range. These most likely originate from zinc nitrate hydroxide family compounds, which crystallise in a monoclinic structure [33]. No distinct reflections associated with Eu-containing phases were detected.

Additionally, a slight shift in the reflections toward higher 2θ angles is visible in the ZnO OVAL samples. The mean crystallite sizes (MCS), calculated using the Scherrer equation based on the (002) reflection, were estimated at approximately 122 nm for ZnO LONG and 52 nm for the ZnO OVAL.

#### 3.1.2. Morphological Characterisation via Scanning Electron Microscopy (SEM)

**Figure 4:**
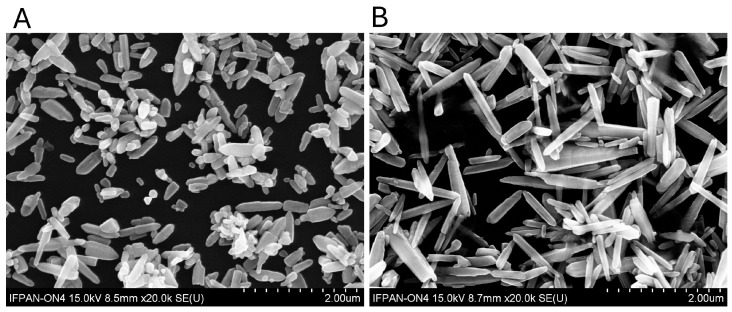
SEM images of ZnO NPs synthesised via microwave-assisted hydrothermal method using different zinc salts. ZnO OVAL (**A**) obtained from anhydrous zinc acetate and ZnO LONG (**B**) synthesised from zinc and europium nitrates.

**Figure 5:**
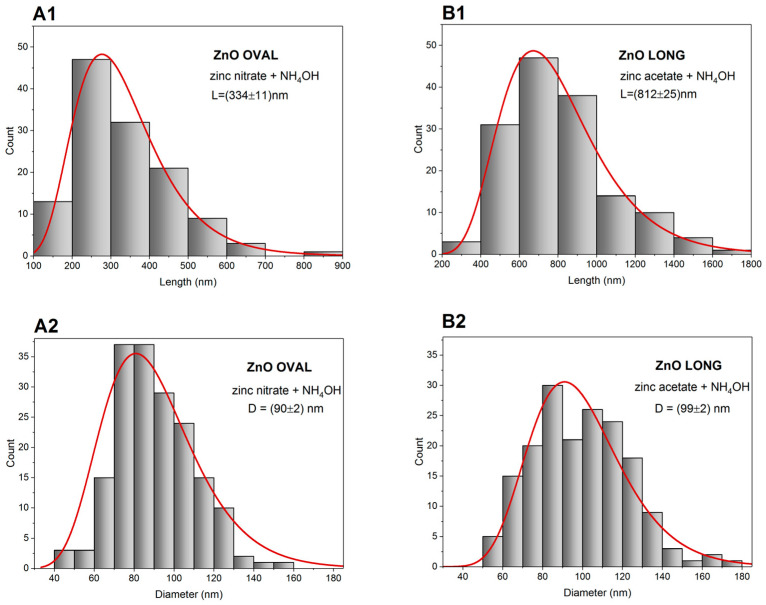
Size histograms with fitted lognormal distribution curves for ZnO NPs dimensions based on SEM image analysis. (**A1**,**A2**)—the length and diameter distributions of ZnO OVAL NPs, respectively; (**B1**,**B2**)—correspond to the length and diameter of ZnO LONG NPs. The mean particle length for ZnO OVAL nanoparticles was determined without considering twin structures. The arithmetic mean nanoparticle lengths are given along with the standard deviation.

The morphology of the synthesised ZnO NPs was investigated using SEM, as shown in Figure 4. ZnO OVAL NPs, synthesised from zinc nitrate and aqueous ammonia solution (Figure 4A), exhibit an oval-like morphology, characterised by a lower shape factor (~3.7) compared to the ZnO LONG, which is derived from zinc acetate. Their average size is also smaller than ZnO LONG, with a mean length of (334 ± 11) nm (Figure 5(A1)) and a mean diameter of D = (90 ± 2) nm (Figure 5(A2)). A more diverse and heterogeneous morphology is also observed in ZnO OVAL. In addition to isolated nanoparticles, twin structures formed by the fusion of smaller crystals are present.

In contrast, ZnO LONG NPs synthesised from anhydrous zinc acetate (Figure 4B) exhibit a well-defined hexagonal prism morphology, with a predominant elongation along the c-axis. Based on direct image analysis, the average length and diameter of the nanoparticles were determined to be L = (812 ± 25) nm (Figure 5(B1)) and D = (99 ± 2) nm, respectively (Figure 5(B2)). The shape factor, defined as the ratio of length (L) to transverse dimension (D), was approximately eight, indicating an anisotropic crystallisation process that favours growth along the (002) axis. The surfaces of the nanoparticles synthesised from zinc nitrate (ZnO OVAL) are more irregular compared to the well-defined edges of those synthesised from zinc acetate (ZnO LONG).

#### 3.1.3. Elemental Composition Analysis by Energy-Dispersive X-Ray Spectroscopy (EDX)

**Figure 6:**
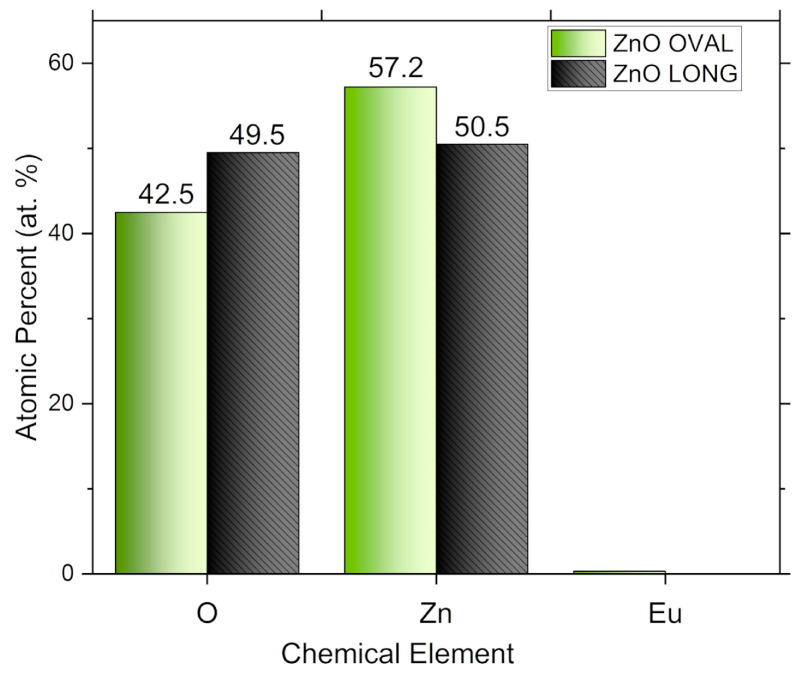
Comparison of atomic percentages of O, Zn, and Eu in ZnO OVAL (ZnO:Eu—green) and ZnO LONG (ZnO—grey) NPs determined by EDX.

The elemental composition of ZnO LONG and ZnO OVAL NPs was analysed using EDX. The atomic percentages of detected elements in samples are presented in Figure 6. In the ZnO LONG, only zinc and oxygen were detected, with atomic percentages of 49.5 at.% and 50.5 at.%, respectively. The composition is consistent with the theoretical stoichiometry of pure ZnO without detectable impurities. In the ZnO OVAL doped with Eu nanoparticles, the EDX spectrum revealed the presence of oxygen (42.5 at.%), zinc (57.2 at.%), and a trace amount of europium (0.32 at.%).

#### 3.1.4. Optical Properties—Cathodoluminescence (CL), Photoluminescence (PL) Measurements and Confocal Microscopy

**Figure 7:**
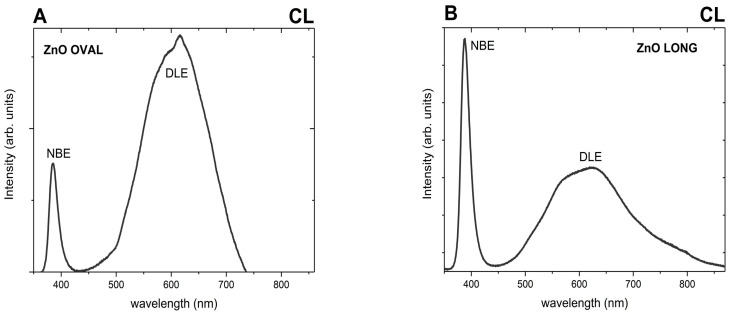
Cathodoluminescence (CL) spectra recorded under electron beam excitation at 15 kV for ZnO OVAL (**A**) and ZnO LONG (**B**) nanoparticles. ZnO OVAL NPs were doped with an additional 0.5 mol% of Eu ions.

The results of room-temperature CL measurements are depicted in Figure 7. Under electron beam excitation at 15 kV, two distinct emission bands are visible for ZnO OVAL (Figure 7A) and ZnO LONG (Figure 7B) NPs. The first, labelled NBE (near band edge), appears in the ultraviolet (UV) region and is connected with excitonic recombination in ZnO [34]. Its maximum is approximately 385 nm for ZnO OVAL and 387 nm for ZnO LONG nanoparticles. The second band, DLE (deep level emission), is significantly broader, extending across the visible range, and is linked to intrinsic ZnO defects. The DLE band appears as a superposition of multiple emission components in both samples.

In the case of the ZnO LONG, the intensity of defect-related luminescence is significantly lower than that of NBE emission, with the calculated ratio of integrated NBE to DLE intensities determined as ~0.3. Nevertheless, the spectral range of the DLE for the ZnO LONG (~450–860 nm) is considerably broader than that of the ZnO OVAL (~420–730 nm), and the DLE band exhibits two distinct local maxima at approximately 570 nm, and 630 nm. The calculated I_NBE_/I_DLE_ ratio for the ZnO OVAL is significantly lower (~0.07), indicating dominant defect-related emission. Furthermore, a narrow emission peak at λ = 614 nm is clearly observed within the broad DLE band of the ZnO OVAL. This feature corresponds to the ^5^D_0_ → ^7^F_2_ transition of Eu^3+^ ions present in the sample.

**Figure 8:**
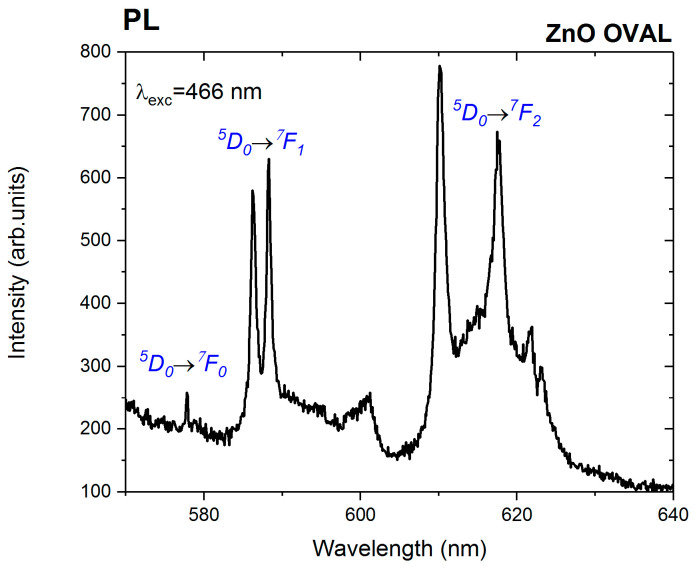
Photoluminescence emission spectrum (λ_exc_ = 466 nm) of ZnO OVAL (Eu-doped) nanoparticles synthesised using zinc nitrate and aqueous ammonia solution.

Doping ZnO with europium ions is generally challenging, due to the significant difference in ionic radii between Zn^2+^ (0.74 Å) and Eu^3+^ (0.95 Å) [35], as well as the discrepancy in their charges. In nanocrystals of this type, Eu^3+^ ions typically localise on the surface or are incorporated into secondary phases crystallising alongside ZnO [36]. To verify whether Eu^3+^ ions were successfully incorporated into the ZnO crystal lattice, emission spectra were recorded at an excitation wavelength of λ_exc_ = 466 nm for oval-shaped ZnO nanoparticles containing 0.5 mol% europium (Figure 8).

Photoluminescence emission spectra recorded under λexc = 466 nm excitation for ZnO OVAL (Eu-doped) NPs revealed distinct narrow emission lines characteristic of Eu^3+^ ions, arising from transitions from the excited state ^5^D_0_ to the ^7^F_J_ (J = 0–4) levels. The presence of a weakly resolved ^5^D_0_ → ^7^F_0_ transition at 577.8 nm indicates that Eu^3+^ ions occupy non-centrosymmetric sites. This spectral line can only appear in low-symmetry point groups such as C_s_–C_1_, C_n_, or C_nv_ (*n* = 2, 3, 4, 6) [37]. The ^5^D_0_ → ^7^F_1_ transition, induced by magnetic dipole interactions, is represented by two spectral components observed at 586 nm and 588 nm. These transitions are generally insensitive to changes in the symmetry of the local coordination environment. The most intense features in the emission spectrum correspond to electric dipole-allowed multiplet ^5^D_0_ → ^7^F_2_, with three lines at 610.2 nm, 617.5 nm, and 621.8 nm. These so-called “hypersensitive” emissions strongly depend on the symmetry of the Eu^3+^ local environment [37]. Broad, low-intensity emission bands are also observed, indicating Eu^3+^ ions occupying multiple, non-equivalent sites.

**Figure 9:**
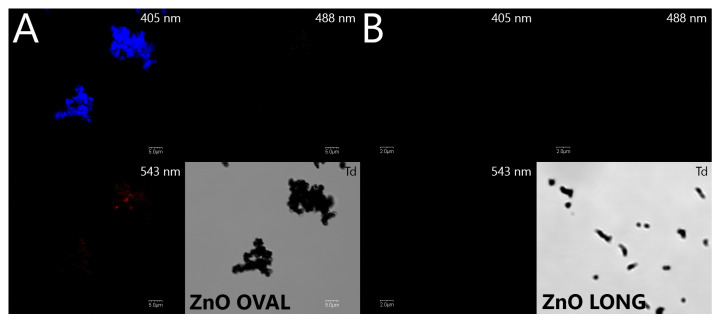
Confocal microscope images of water-dispersed ZnO OVAL (**A**) and ZnO LONG (**B**) NPs showing fluorescence yields at 430–450 nm (for 405 nm excitation), 505–525 nm (for 488 nm excitation) and 560 IF (for 543 nm excitation). Shape of NPs aggregates is visible under transmitted light detector (Td). Visible blue fluorescence for ZnO OVAL is most likely associated with Zn crystal surface defects following Eu embedment in the crystalline matrix. Lens magnification 40×, digital zoom 5×. Confocal microscopy figures were combined in Adobe Photoshop CS6.

Eu ions were introduced as a doping agent for ZnO to induce their fluorescence, hence NPs detection in the embryonic tissues under the confocal microscopy systems that are widely used in biomedical studies. As the ZnO lattice biodegrades, the fluorescence characteristic for Eu-induced ZnO defects disappears [38], allowing the direct confirmation of NPs presence and not the Zn ions effect. As seen in Figure 9, the intensive surface-defect-related fluorescence under the 405 nm excitation was observed in the case of ZnO OVAL NPs, with trace red fluorescence related to Eu ions in the range of 560 IF under excitation of 543 nm (Figure 9A). No fluorescence was observed under any of the available excitation sources for ZnO LONG NPs (Figure 9B).

#### 3.1.5. Results of Zeta Potential Measurements

**Figure 10:**
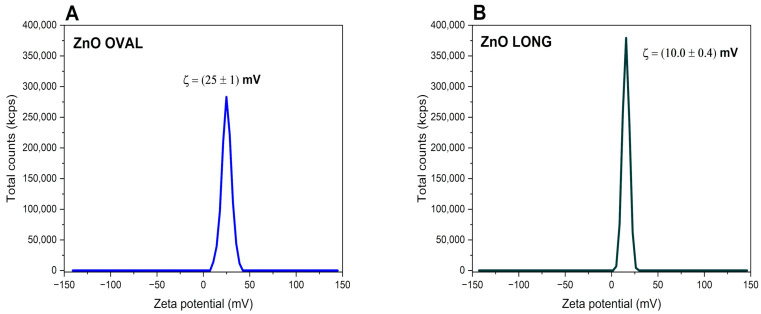
Zeta potential distribution of ZnO OVAL (**A**) and ZnO LONG NPs (**B**).

The zeta potential measurement of ZnO LONG synthesised using zinc acetate was determined to be (10.0 ± 0.4) mV, whereas the ZnO OVAL obtained from zinc nitrates exhibited a significantly higher value of (25 ± 1) mV. These differences indicate a pronounced variation in surface charge characteristics, suggesting distinct colloidal stability profiles for the two materials. The corresponding zeta potential distributions are shown in Figure 10.

The hydrodynamic diameters (Z-average) and polydispersity indices (PDI) of the samples are presented in Table 2. ZnO OVAL exhibited a mean diameter of (369 ± 7) nm with a PDI of 0.1854, indicating a relatively narrow size distribution. In contrast, ZnO LONG had a much larger average hydrodynamic size of (2845 ± 95) nm and a similar PDI of 0.2008.

### 3.2. Results—Biological Study

#### 3.2.1. Cell Viability Assay

**Figure 11:**
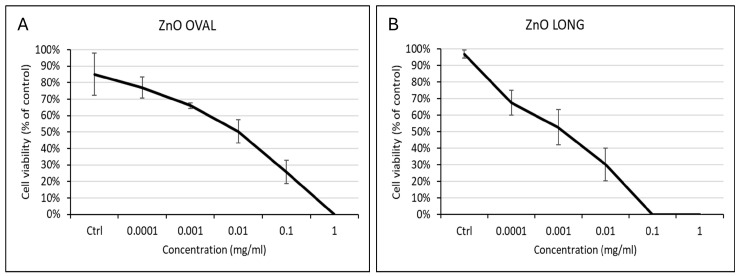
Cell viability of primary chicken embryo fibroblasts (CEFs) as % of viable cells 24 h after administration of ZnO OVAL (**A**) and ZnO LONG (**B**) at different concentrations. For CTRL, PBS aliquots were added to the culture medium.

The cell viability test showed differences depending on the shape of the used NPs. With ZnO OVAL, CEFs viability dropped to 50% for a concentration of 0.01 mg/mL (Figure 11A). For ZnO LONG the decrease in the CEFs viability to 50% occurred at a concentration of 0.001 mg/mL (Figure 11B), indicating that they are higher in vitro toxicity.

#### 3.2.2. Zn Concentration in Embryo and Egg White After Administration of Nanoparticles Measured by Atomic Absorption Spectrometry (AAS)

**Figure 12:**
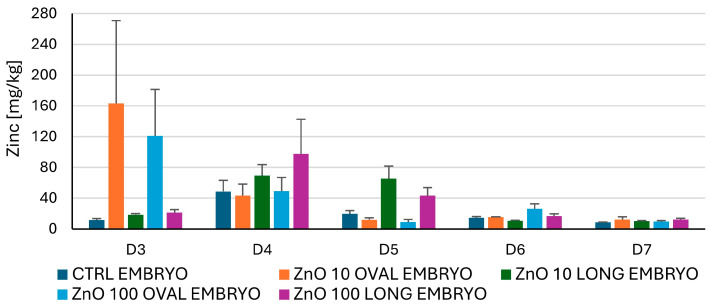
Average zinc concentration in chicken embryos from days 3 to 7, after administration of a single dose of 10 µg/mL (ZnO 10) or 100 µg/mL (ZnO 100) of ZnO OVAL or ZnO LONG. Error bars represent standard deviations (SD).

AAS was used to indirectly quantitatively measure the distribution of ZnO NPs in the egg after administration to the air chamber. The concentrations in egg white did not vary from the CTRL and did not exceed 5 mg/kg, the average Zn concentration in the egg, indicating no significant shift in Zn concentration in the egg following NPs application. The mean results for zinc concentration in embryos are presented in Figure 12. Due to high individual variability in Zn content following the application of ZnO OVAL on day three, no statistical significance was found; however, the trend (*p* < 0.1) was observed for both NPs concentrations (10 and 100 µg/mL) for ZnO OVAL between days three and four, and between ZnO OVAL and ZnO LONG at day three (for both NPs concentrations).

Despite the lack of statistical significance, the observed difference in zinc concentration in embryos, which depends on the shape of NPs at day three (trend), can also be observed at days four and five, where ZnO LONG achieve higher concentrations within the embryo (trend, *p* < 0.1 at day five). Also, the decrease in Zn levels to CTRL values was observed with a two day shift between ZnO OVAL (at day four for both concentrations) and ZnO LONG (at day six for both concentrations).

#### 3.2.3. Fluorescence in Embryo Tissues

**Figure 13:**
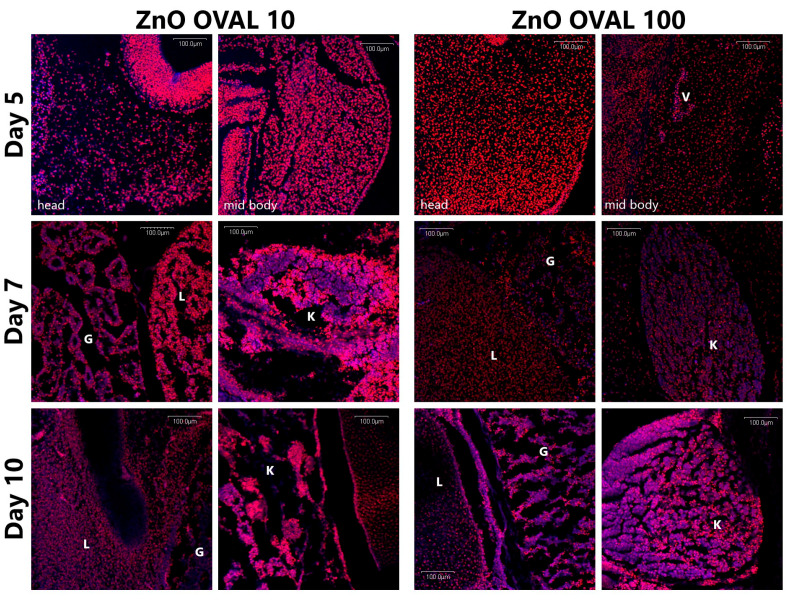
Confocal microscopy image of chicken embryo tissues following administration of ZnO OVAL NPs at concentrations 10 µg/mL (ZnO OVAL 10) and 100 µg/mL (ZnO OVAL 100) at days 5, 7, and 10 of incubation. Lens magnification 20×. Red fluorescence corresponds to nuclei stained with 7-AAD, while blue fluorescence indicates ZnO OVAL NPs. L—liver, K—kidney, G—intestine, V—vessel. Confocal microscopy figures were combined in Adobe Photoshop CS6.

By incorporating Eu doping into ZnO OVAL nanoparticles, the presence of nanoparticles in embryonic tissues could be directly confirmed using confocal microscopy (Figure 13). In day five embryos, a higher level of ZnO OVAL NPs was observed following exposure to a dose of 10 µg/mL, compared with 100 µg/mL. In day seven embryos, NPs were mainly detected in intestinal (G) and kidney (K) tissues, with comparatively lower presence in the liver (L). In day ten embryos, ZnO OVAL NPs were observed in the liver (L), intestine (G), and kidney (K) tissues for both administered doses.

#### 3.2.4. Activity of Superoxide Dismutase (SOD)

**Figure 14:**
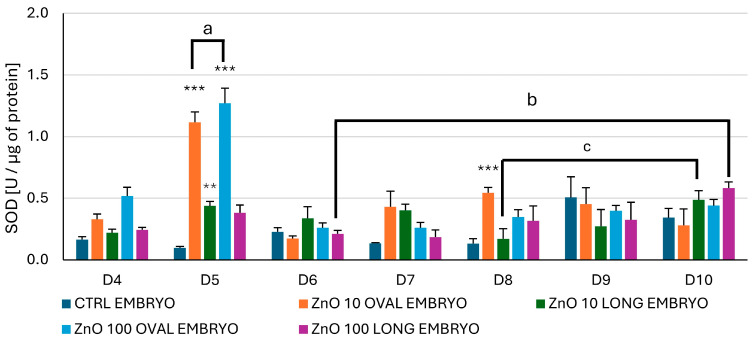
Superoxide dismutase (SOD) activity in chicken embryos from day 4 to 10 following administration of a single dose of 10 µg/mL (ZnO 10) or 100 µg/mL (ZnO 100) of ZnO OVAL or ZnO LONG nanoparticles. Error bars represent standard deviations (SD). A total of 1 U—amount of enzyme extract that inhibited formazan formation by 50%. Asterisks indicate significant variation vs. CTRL of that day; ** *p* < 0.01; *** *p* < 0.001. Letters indicate statistically significant differences: a—doses that are statistically significant compared with the other experimental days; b, c—statistical significance between indicated days.

The activity of SOD changed significantly vs. CTRL during days five and eight of the incubation (Figure 14). On day five, after administration of the ZnO OVAL, a highly significant increase (*p* < 0.001) in SOD activity was observed at both NPs concentrations and for ZnO LONG at 10 µg/mL. Also, there was a significant (*p* < 0.01) difference in the SOD activity (marked as a and b) between ZnO OVAL 10 and 100 µg/mL. For ZnO LONG, the significant increase in the SOD activity was observed between days six and ten (marked as c) for higher NPs concentration (100 µg/mL), and between days eight and ten for lower concentration (10 µg/mL).

#### 3.2.5. Lipid Peroxidation Measured as the Content of Malondialdehyde (MDA) in the Embryo

**Figure 15:**
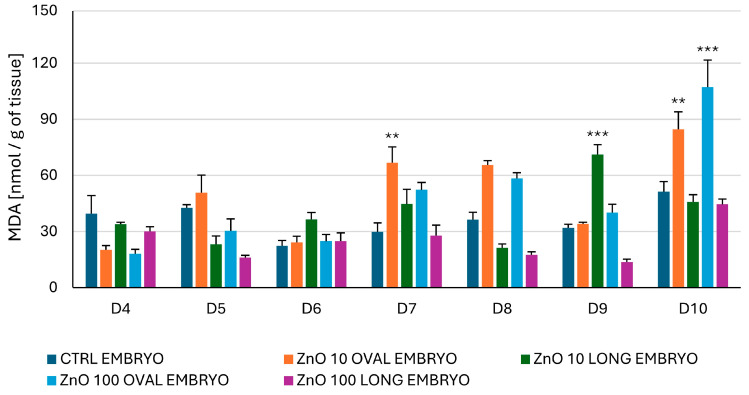
Malondialdehyde (MDA) content in chicken embryos from days 4 to 10 following administration of a single dose of 10 µg/mL (ZnO 10) or 100 µg/mL (ZnO 100) of ZnO OVAL or ZnO LONG nanoparticles. Error bars represent standard deviations (SD). Asterisks indicate significant variation vs. CTRL of that day; ** *p* < 0.01; *** *p* < 0.001.

The level of lipid peroxidation in the embryo after administration of ZnO OVAL at both concentrations (10 and 100 µg/mL) shows a tendency to increase from day five until the last test day, (a drop to CTRL level was observed at day nine) with a significant increase vs. CTRL (*p* < 0.01) observed at days seven and ten for 10 µg/mL, and a highly significant increase (*p* < 0.001) at day ten for 100 µg/mL (Figure 15). For ZnO LONG at a concentration of 10 µg/mL, a tendency of increased lipid peroxidation was observed from day six with a highly significant (*p* < 0.001) increase vs. CTRL observed at day nine (Figure 15). In contrast, for ZnO LONG at a concentration of 100 µg/mL a tendency of lipid oxidation at the level of CTRL or lower is observed (no statistical significance nor trend).

#### 3.2.6. Content of Carbonylated Proteins (CP) in the Embryo

**Figure 16:**
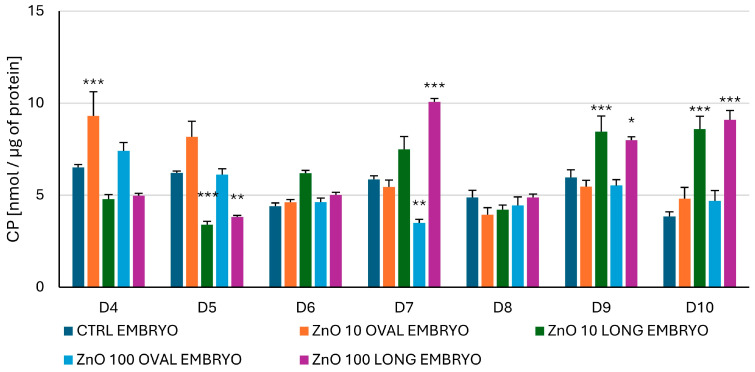
Carbonylated proteins (CP) content in chicken embryos from days 4 to 10 following administration of a single dose of 10 µg/mL (ZnO 10) or 100 µg/mL (ZnO 100) of ZnO OVAL or ZnO LONG nanoparticles. Error bars represent standard deviations (SD). Asterisks indicate significant variation vs. CTRL of that day; * *p* < 0.05; ** *p* < 0.01; *** *p* < 0.001.

Significant variations vs. CTRL in the amount of CP were already observed from day four of incubation (Figure 16). For ZnO OVAL, only significant changes were observed for 10 µg/mL concentration; a highly significant (*p* < 0.001) increase was observed on day four after NPs administration, followed by a trend (*p* < 0.1) increase at day five. For 100 µg/mL concentration of ZnO OVAL, content of carbonylated proteins oscillated around CTRL for all days, except day seven, when a significant (*p* < 0.01) drop was observed. In the case of ZnO LONG, a highly significant decrease (*p* < 0.001) in the amount of carbonylated proteins was observed on day five, and highly significant increases (*p* < 0.001) at days nine and ten. For ZnO LONG 100 µg/mL, a significant drop at day five (*p* < 0.01) was followed by a highly significant increase at day seven (*p* < 0.001), a significant (*p* < 0.05) increase at day nine, and a highly significant (*p* < 0.001) increase at day ten.

#### 3.2.7. Whole Genomic Microarray and Real-Time PCR

**Figure 17:**
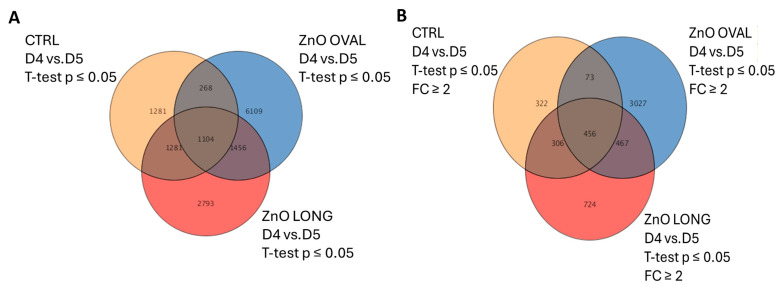
Number of all transcripts whose expression has changed statistically significantly (Moderated T-test *p* ≤ 0.05, FDR < 0.05), shown in the Venn diagram (**A**) and after considering changes only in transcripts whose fold change (FC) ≥ 2 (**B**). Changes for ZnO OVAL are shown in blue, while changes for ZnO LONG are shown in red.

To check the impact of the ZnO NPs at the genomic level, an analysis using microarrays was performed according to the diagram presented in Figure 2. Chicken embryos were treated with 10 µg/mL ZnO OVAL or ZnO LONG and incubated for four or five days. Our main goal in this experiment was to check shape-specific changes caused by NPs, and as a result, we observed changes in the expression of 6109 transcripts specific only to ZnO OVAL and a change in the expression of 2793 transcripts for ZnO LONG (Figure 17A). After considering transcripts which fold change (FC) ≥ 2, we were left with 3027 transcripts changing between day four and five for ZnO OVAL, and 724 transcripts for ZnO LONG (Figure 17B). In the next step of the analysis, transcripts were selected that were assigned to specific genes and then, using the Reactome pathway database, we investigated which pathways they were assigned to. For ZnO OVAL, 1487 transcripts were assigned to specific genes, and 1108 of these were assigned to pathways in the Reactome database. For ZnO LONG, 548 genes were identified, of which 358 were assigned to pathways. For both shapes of ZnO NPs, the main changes occurred in genes related to signal transduction and energy metabolism.

Comparing day four vs. day five, for ZnO OVAL, there was an increase in expression for 38 genes and a decrease in expression for 180 genes associated with signal transduction (including tyrosine kinase pathways). In contrast, for ZnO LONG, there was a respective increase in expression for 50 genes and a decrease in expression for 33 genes. For changes in gene expression related to energy metabolism, in ZnO OVAL, an increase in expression was observed for 15 genes, mainly related to metabolic changes in lipids, nucleotides, vitamins, and oxidative stress; the expression of 151 genes was downregulated. For ZnO LONG, the expression of only 38 genes was increased, while the expression of 36 genes was decreased.

**Figure 18:**
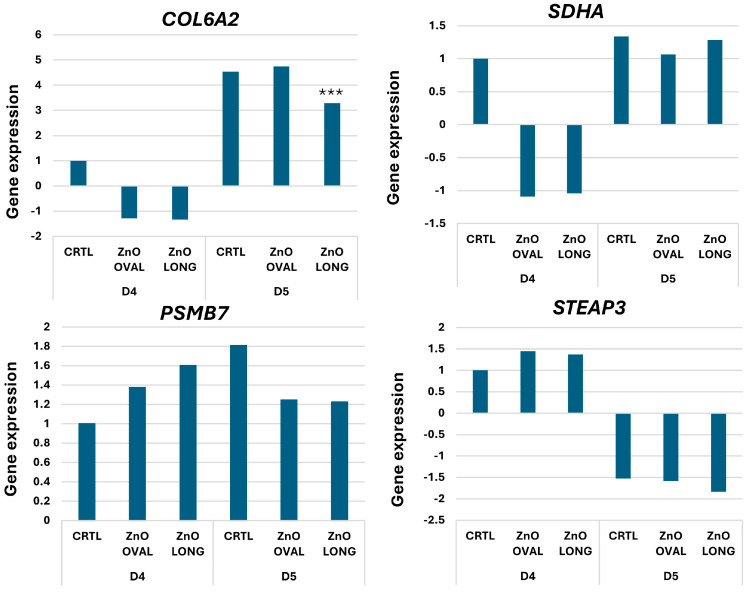
Relative quantification of *COL6A2*, *SDHA, PSMB7*, *STEAP3* gene expression. Asterisks indicate high significance vs. CTRL; *** *p* < 0.001.

Real-time PCR analysis was performed to confirm the validity of microarray results. Three genes were selected for confirmation: *COL6A2*, *STEAP3,* and *PSMB7* (Table 3). Moreover, during the tests for reference genes, we observed an interesting result for *SDHA,* which is also presented (Figure 18). A statistically significant drop was only observed for *COL6A2* for ZnO LONG at day five vs. CTRL (*p* < 0.001). Expressions of all chosen genes correlated with the microarray results.

Expression for *COL6A2* on day four was lower than in the CTRL, and at day five was increased compared to day four, consistent with the microarrays. In the case of the *STEAP3* gene, although the results obtained by the real-time PCR method are not statistically significant, they show the same tendency of expression change as in the case of the microarray. The *STEAP3* expression was higher on day four than day five in all studied groups. For expression of the *STEAP3* gene, despite observing a rather high fold change based on the microarray results, these changes were not as high for real-time PCR, and neither were they statistically significant. For the *SDHA* gene, a change in expression relative to the control was only observed for day four, without statistical significance. On the following day, *SDHA* expression levels were similar to the control. For the *PSMB7* gene, no statistically significant changes in expression levels were observed. However, an elevated expression level was noted on day four, following the administration of both types of nanoparticles compared with the control, with ZnO LONG nanoparticles exerting a greater effect on that day. In contrast, on day five, a decrease in expression relative to the CTRL was observed.

## 4. Discussion

### 4.1. Physicochemical Properties

The structural, morphological, and optical differences observed between ZnO OVAL and ZnO LONG NPs reflect the impact of synthesis conditions and Eu doping on ZnO nanoparticle development. As revealed by X-ray diffraction (XRD), the shift in XRD peaks toward higher angles in Eu-doped ZnO OVAL is notable. This behaviour is unusual, as Eu^3+^ ions have a larger ionic radius (~0.95 Å) than Zn^2+^ (~0.74 Å) [35], which would typically provide an increase in interplanar spacing, leading to an expansion of the crystal lattice and a shift in the diffraction peaks towards lower angles [39,40]. However, the observed shift toward higher angles suggests lattice compression, which agrees with the law of Braggs. Similar effects have been reported previously [39,41] and were attributed to the presence of an additional Eu_2_O_3_ phase (not detected in our data), to Eu ion segregation at the nanoparticle surface or incorporation of Eu^3+^ ions into interstitial positions [42]. Even trace amounts of secondary phase, which may remain undetectable by XRD, may influence the main ZnO reflections by inducing a local stress. In addition, doping with a non-isovalent element like Eu^3+^ may trigger charge compensation mechanisms, such as the formation of anion vacancies, leading to local lattice distortions [43]. The synthesis route may also influence the shift, as the two sample types were prepared using different precursors and reaction conditions. These variables can significantly impact the crystallisation pathway, morphology, and defect density, all of which affect the final structural parameters. It is noteworthy that despite numerous attempts, the incorporation of Eu ions within the structural lattice of ZnO LONG NPs proved, hitherto, unsuccessful. The choice of precursor has a clear effect on nanoparticle growth. It is generally observed that nanoparticles synthesised from zinc nitrate exhibit more significant morphological defects [27]. In our case, zinc acetate favours the formation of well-defined hexagonal ZnO nanostructures. In Figure 3 there are observed, apart from zinc oxide reflexes, heavily broadened reflexes located at ca. 2theta 19.5; 22.3; 25.5 degrees. Appearance of these features is related to ZnO crystallization mechanism starting from Zn(CH_3_COO)_2_. Hydrolysis of this precursor results in occurrence of intermediate Zn_4_O(CH_3_COO)_6_ and exhaustion of vaporous acetic acid. In the second step, further hydrolysis of this compound leads to crystallization of wurtzite type ZnO and exhalation of gaseous acetic acid and carbon dioxide. Presence of CO_2_ and basic environment causes synthesis of secondary products family: zinc carbonate hydroxide complexes. In the stoichiometric unit there are variable numbers of CO_3_^¯^ and HO^¯^ ligands, also hydration level may change. Thus precise determination of phases present in this sample is difficult, as reflexes from different complexes (e.g. Zn_4_CO_3_(OH)_6_·H_2_O) exhibit different 2 theta positions. Additionally, the presence of Eu ions in the zinc nitrate-based sample is also an important factor. As noted earlier, Eu^3+^ ions have a larger ionic radius than Zn^2+^ ions, and their incorporation into the crystal lattice can induce local stresses, growth disturbances, and the associated formation of defects. Previous studies [44] have shown that introducing Eu^3+^ ions into the ZnO matrix can lead to planar defects along the (0001) plane, interfering with the growth process and reducing nanoparticle length. In agreement with these reports, we observed a morphological transformation of the Eu-doped ZnO nanoparticles toward a more oval geometry, accompanied by a reduction in shape ratio to ~4 (ZnO OVAL). This change may also be influenced by differences in the chemical nature of the synthesis precursors used in hydrothermal synthesis.

The noticeable discrepancy between the sizes obtained from SEM (Figure 4) and XRD measurements may suggest that the objects observed in the SEM images are composed of smaller crystallites and form agglomerates or aggregates. This is especially evident for ZnO OVAL, which exhibited irregular morphologies and signs of fusion between particles. Additional insights are provided by dynamic light scattering (DLS) measurements of the hydrodynamic size distribution. Generally, obtained hydrodynamic sizes are larger compared to both XRD and SEM.

The ZnO LONG nanoparticles exhibited a significantly larger average hydrodynamic diameter D = (2845 ± 95) nm compared to ZnO OVAL (369 ± 7) nm. It is important to note that the DLS technique assumes a spherical particle geometry and does not adequately account for shape anisotropy. This results in an overestimation of the size of long nanoparticles due to their slower translational diffusion. Consequently, the unusually large DLS size recorded for ZnO LONG may not reflect the aggregation of particles in aqueous suspension, but point towards the inherent limitations of the measurement technique when applied to long, anisotropic structures. The low polydispersity index (PDI), which is below 0.3 for both samples, indicates that the particles were well-dispersed at the time of measurement. Generally, the hydrodynamic diameters are larger than the transverse sizes obtained directly from SEM images, owing to the presence of hydration layers, surface-bound ions, or aggregation in suspension.

The results of energy-dispersive X-ray spectroscopy (EDX) support the successful synthesis of stoichiometric ZnO LONG, with elemental ratios closely matching the theoretical Zn:O = 1:1 value. In the case of the Eu-doped ZnO OVAL sample, the presence of europium was confirmed at an atomic concentration of 0.32%, which is in reasonable agreement with the intended doping level of 0.5 mol%. This minor deviation falls within the expected error range for EDX quantification at low doping levels. These findings support the spectroscopic evidence for Eu^3+^ incorporation, as indicated by the appearance of characteristic emission lines associated with Eu^3+^ transitions and corresponding changes in the optical properties.

In both types of ZnO NPs, the DLE band appears as a superposition of multiple emission components. This spectral heterogeneity reflects the involvement of multiple defect centres, which result from both the instability of point defects and their diverse structural forms. There is also considerable variation in the reported energy levels that individual point defects occupy within the band gap. Notably, the interpretation of the defect-related luminescence band remains highly debated, and its exact origin has yet to be definitively established [34]. The yellow emission band (~570 nm), particularly prominent in spectra of ZnO LONG, is often attributed to acceptor-like defects and may be associated with a deep acceptor level, related to interstitial oxygen (O_i_) [45]. Other sources report that, in materials synthesised via hydrothermal methods, emission in this spectral range may indicate the presence of zinc hydroxide (Zn(OH)_2_) [46], which can form in the reaction solution and adsorb onto the surface of the nanoparticles. In contrast, the red emission observed in the spectra of both samples may be attributed to interstitial zinc (Zn_i_) [47], O_i_, or excess oxygen [48]. The near-infrared (NIR) emission, which in the case of elongated nanoparticles extends up to ~900 nm, is tentatively assigned to donor–acceptor pair transitions, involving oxygen vacancies (V_o_) and zinc vacancies (V_Zn_), or connected to transitions involving shallow electron traps and deeply bound hole states associated with interstitial oxygen defects [49].

The significantly lower I_NBE_/I_DLE_ ratio (~0.07) obtained from cathodoluminescence spectra of ZnO OVAL:Eu samples is consistent with structural irregularity and higher surface defect density. According to our previous observations, [27] ZnO NPs synthesised with zinc nitrate and NH_4_OH as alkalizing agents are characterised with high contribution of the DLE band. Simultaneously, the reduction in NBE emission intensity may be attributed to the efficient trapping of charge carriers by these defects and enhanced non-radiative recombination. The incorporation of Eu ions may have promoted to an increased structural defect density, including for example, interstitial zinc [50]. It is also proposed that, in Eu-doped ZnO nanoparticles, the red emission may partially originate from intra-4f shell transitions associated with Eu_zn_–O_i_ defect complexes [50]. A narrow emission peak prominent at 614 nm over the broad defect-related cathodoluminescence spectrum corresponds to the hypersensitive ^5^D_0_ → ^7^F_2_ transition of Eu^3+^ ions, confirming their presence in ZnO OVAL, also visible as a trace red emission in confocal microscopy (Figure 9). The Eu^3+^ ion, with its electronic configuration [Xe] 4f^6^, can be considered a spectroscopic probe, allowing for analysis of the local symmetry around the ion within a given host matrix. This is partly because different point groups impose different selection rules and allow for a varying number of transitions between two terms. A decrease in local symmetry relaxes the Laporte selection rules, thereby permitting a greater number of electric dipole transitions [37]. For the ZnO OVAL, a weak visible single emission line assigned to the ^5^D_0_ → ^7^F_0_ transition was observed, along with two sharp lines from the ^5^D_0_ → ^7^F_1_ group and three distinct peaks belonging to the ^5^D_0_ → ^7^F_2_ group. The number and distribution of these emission lines indicate that a significant fraction of Eu^3+^ ions are located at sites exhibiting C_3v_ point group symmetry [37].

In addition to the characteristic sharp emission lines, broad bands of a significantly lower intensity were also detected. These features suggest the presence of Eu^3+^ ions in multiple non-equivalent environments, including surface sites, distorted regions of the lattice such as grain boundaries, or structural defects like vacancies, related to the dominant blue emission observed under 405 nm excitation (Figure 9). Nevertheless, the dominant environment of Eu^3+^ ions appears to be associated with C_3v_ symmetry, indicating their preferential substitution at Zn^2+^ sites within the wurtzite ZnO lattice, while retaining the local coordination geometry.

The observed differences in zeta potential values between ZnO LONG and ZnO OVAL can be attributed to chemical composition surface-defect density variations and distinct morphological characteristics. ZnO LONG synthesised from zinc acetate exhibits an elongated, needle-like morphology, while the ZnO OVAL displays a more isotropic, oval shape. It is important to know that zeta potential measurements performed using electrophoretic light scattering, similar to the DLS measurements, are based on the assumption of spherical particle geometry. Anisotropic structures, such as elongated particles, tend to rotate in the applied electric field and can present heterogeneous surface charge distributions. These phenomena disturb electrophoretic (ELS) mobility measurements and often underestimate the zeta potential. Therefore, the relatively low zeta potential recorded for the extended ZnO nanoparticles (10.0 ± 0.4) mV may be partially attributed to these geometric effects, rather than reflecting a genuinely lower surface charge density or reduced colloidal stability. It is known that nanoparticles exhibiting a neutral or near-neutral surface charge (zeta potential ≤ ±10 mV) are typically unstable in dispension, making them susceptible to agglomeration, flocculation, or coagulation over time [51]. In contrast, the more symmetric ZnO OVAL (+25 ± 1 mV) better satisfy the assumptions underlying the ELS model, making their measured zeta potential more representative of the actual electrostatic surface potential. Furthermore, Eu doping may promote the formation of surface-related defects, such as oxygen vacancies, which may impact the observed increase in surface charge. This is consistent with SEM observations, where Eu-doped ZnO OVAL particles displayed a less homogeneous surface texture than their undoped counterparts and cathodoluminescence spectra, with reduced I_NBE_/I_DLE_ ratio compared to pure ZnO sample. These findings suggest that both morphological anisotropy and dopant-induced surface states play a crucial role in determining the colloidal behaviour and electrostatic properties of ZnO-based nanomaterials.

### 4.2. Biological Study

A strong increase in zinc concentration in chicken embryos is seen for the ZnO OVAL on day three of incubation, followed by a rapid decrease on day four to the level of the control (Figure 12). The lower zinc concentration for the higher dose on day three of incubation may be due to increased NPs aggregation, affecting their penetration into the embryo. For both NPs concentrations (10 and 100 µg/mL), zinc levels in the embryos drop to control levels on day four, following incubation. This may be attributed to the activation of NPs removal systems in the developing embryo and, surprisingly, coincides with the beginning of kidney formation and its functional development in chicken embryos [52,53]. Notably, since previous studies concerning mammalian models showed the predominant role of liver and bile in the NPs recirculation and removal from the organism [3,54,55,56,57]. This may indicate that in birds, the NPs removal occurs predominantly through the kidneys, or it is an embryo-specific factor. Regardless, this is a new development and requires further study. These observations are supported by the confocal microscopy imaging, where ZnO-OVAL-related blue fluorescence diminishes over time; however, the high levels on NPs are continuously observed within kidney tissues (Figure 13). Furthermore, confocal studies show differences between NPs permeability to the embryo tissues at different concentrations. Lower ZnO OVAL concentration of 10 µg/mL (Figure 13 ZnO OVAL 10) permeated embryonic tissues faster, which is most probably associated with higher aggregation of NPs at the concentration of 100 µg/mL, resulting with inability to cross cell membrane and tissue barriers (note the uneven NPs distribution at 100 µg/mL and high NPs concentration in the tissues surrounding blood vessel—Figure 13 ZnO OVAL 100). The influence of concentration on nanoparticle aggregation has also been observed in other studies [58,59].

Comparing AAS results for ZnO OVAL and ZnO LONG, a relative shift in the increase in zinc concentration in the embryos and a subsequent drop in Zn levels can be observed for ZnO LONG on day four and day six, respectively. This may indicate that nanoparticles with an elongated shape manifest a lower ability to migrate within the egg, both into and out of the embryo. Also, the relatively lower zeta potential value may affect the dispersal of those NPs within a predominantly negatively charged environment of the egg. The importance of the shape and structure of the ZnO NPs on their biological properties is shown by the cell viability assay, where ZnO LONG exhibited higher toxicity to the primary CEFs with CD50 at 0.001 mg/mL, compared to 0.01 of ZnO OVAL. However, those results were not confirmed by in ovo experiments, where no effect of NPs on embryo viability was observed, further supporting the different dynamics of ZnO OVAL and ZnO LONG diffusion in the egg and their trafficking in the tissues of an embryo.

The results for the Zn concentration in the egg white did not show any variation vs. CTRL, nor any statistical significance, and measured Zn concentrations did not exceed 5 mg/kg. Compared to the average Zn content in the egg [60], the applied doses of NPs did not alter the overall Zn pool nor were they expected to significantly alter Zn turnover. Therefore, a relationship between the drop in Zn levels in embryos and egg white results was not expected and not demonstrated. Importantly, the AAS does not provide information about the form of zinc, but only about its concentration in the examined volume/mass. This means that the results include zinc in both nanoparticle and ionic form. However, as demonstrated in the confocal microphotographs, the presence of NPs-related fluorescence clearly indicates the presence of ZnO OVAL NPs in the majority of embryonic tissues (Figure 13). Also, in a study with mice, where Eu-doped ZnO NPs were administered, the organism was quickly able to regulate zinc levels by removing its excess, but at the same time, NPs-related fluorescence was visible in histological samples [3,54,55,56]. Comparing AAS results and confocal microphotographs indicates that embryonic tissues are also able to regulate Zn content, regardless of its formulation (compare Figure 12 and Figure 13).

One of the consequences of elevated Zn content in the tissues is the increase in the level of ROS [7]. Following lipid peroxidation and accumulation of carbonylated proteins, as well as changes in SOD, may indicate the oxidative stress and may be the first indication of organism reaction to metal oxide NPs. Surprisingly, our study showed that only the levels of CP coincided with the elevated Zn content in the chicken embryo (Figure 16). In general, both lipid peroxidation, indicated by increasing levels of MDA (Figure 15), and CP levels (Figure 16), increased gradually from day seven to day ten, supporting the theory that, like in mice, the Zn levels alone may not reflect true accumulations of ZnO NPs in the tissues. The activity of SOD is one of the markers of oxidative stress, since enzymes from this family are taking part in the transformation of superoxide radical anion to H_2_O_2_. Comparing SOD activity (Figure 14) with the AAS results for both ZnO NPs, a significant peak of dismutase activity coincides with the drop of Zn levels to the CTRL, suggesting that the trafficking NPs and associated energy requirements and signal transduction (as shown by the microarray study) may induce the oxidative stress more than just the presence of the ZnO NPs in the embryonic tissues. The early oxidative stress event associated with the higher dynamics of ZnO LONG trafficking and carbonylated protein content appears to be counteracted by a strong antioxidant response, as evidenced by the marked increase in SOD activity on day five (Figure 14). By day six, both SOD activity and protein carbonyl levels return to the control values, suggesting effective activation and mitigation of the oxidative imbalance.

The *COL6A2* gene, selected for analysis, encodes the α2(VI) chain of collagen VI (COL6), which is a major structural component of the extracellular matrix and is highly expressed in the brain [61]. COL6 has been shown to play an important role in the central nerve system, where its deficiency leads to impaired regulation of autophagy, increased susceptibility to oxidative stress, and spontaneous neuronal apoptosis [62]. On day five, after administration of ZnO LONG nanoparticles, a statistically significant decrease in *COL6A2* gene expression was observed compared to CTRL on that day (Figure 18), which may indicate early disruption of extracellular matrix integrity associated with oxidative stress. A tendency of increasing MDA content was also detected from six days after administration (Figure 15) and associated tendency of increased accumulation of CP (Figure 16) were documented in the current study, which may suggest a progressive accumulation of peroxidative damage.

The *SDHA* gene encodes the major catalytic subunit of the enzyme succinate dehydrogenase [63]. Although the reduction in *SDHA* gene expression observed on day four was not statistically significant, it reflects a subtle but consistent trend (Figure 18), potentially indicating an early disturbance in mitochondrial function and elevated ROS production [64]. Other studies have shown the correlation between a decrease in the expression of one of the *SDH* subunits and an increase in SOD activity [65]. The decrease in SDHA gene expression in our study coincides with a marked increase in SOD activity on day five (Figure 14), particularly in embryos treated with ZnO OVAL. Both tested NPs concentrations triggered a comparable rise in SOD activity, suggesting that even low concentrations of ZnO NPs can activate antioxidant defences. The timing of this antioxidant response may reflect an early cellular reaction to increased ROS levels [66], likely resulting from nanoparticle exposure during sensitive stages of embryonic development.

*PSMB7* encodes the β-type subunit seven of the 20 S proteasome core, which is responsible for the proteolytic activity during the degradation of damaged, misfolded, or non-functional proteins. Maintaining protein homeostasis, particularly in response to oxidative damage, relies heavily on the proteasome. It plays an essential role in recognising and removing dysfunctional proteins [67]. In the current study, a trend toward an increased expression of the *PSMB7* gene was observed on day four, following the administration of both ZnO OVAL and ZnO LONG nanoparticles (Figure 18). Although not statistically significant, this upward trend may reflect an early proteasomal response to elevated levels of damaged proteins, as indicated by the accumulation of CP, particularly in embryos exposed to ZnO OVAL (Figure 16). The oxidative stress-associated increase in protein carbonylation suggests ongoing peroxidative damage, which may require enhanced proteolytic clearance via the ubiquitin–proteasome pathway.

Lipid peroxidation is a marker of oxidative stress that can have a significant impact on the developing embryo. As can be observed, the increase in peroxidation (MDA) levels is more characteristic to ZnO OVAL exposure than to ZnO LONG (Figure 15). This, especially when correlated with the AAS and microarray results, may suggest that trans-membrane trafficking of ZnO NPs may contribute to the increased lipid peroxidation.

The differences in the markers of the oxidative stress observed between ZnO OVAL and ZnO LONG (see Figure 14, Figure 15 and Figure 16) and differences in their impact on the gene expression (Figure 17 and Figure 18) indicate the strong link between NPs morphology and their impact on the organism, which is often neglected by researchers. We proved that the morphology of ZnO NPs had a significantly higher influence on the timing and dynamics of oxidative damage in the developing embryo than the tenfold increase in NPs concentration. Furthermore, with the increase in the concentration of NPs, their aggregation also increases, which was also shown by the discrepancy between the sizes obtained from SEM, hydrodynamic size and XRD (compare Figure 3, Figure 4 and Figure 5 and Table 2). The clear difference in tissue permeability between concentrations of ZnO OVAL NPs was shown on confocal microphotographs (Figure 13). At a higher dose, the NPs may be easier to aggregate into larger structures, which may hinder their ability to permeate into the tissues and cells. AAS also may false-positively increase the Zn (NPs) concentrations in the tissues at higher NPs concentrations, as it does not differentiate between NPs present inside the cells and those present in circulation and loosely associated with erythrocytes (see vicinity of the vessel at Figure 13—ZnO OVAL 100, day five).

The overall level of changes at the genomic level presented by the results of microarray study was overwhelming. Changes in the expression of 1487 identified genes (1108 assigned to pathways) for ZnO OVAL and 548 identified genes (358 assigned to pathways) for ZnO LONG shows not only a strong dependence on the shape of the nanoparticles used, but most crucially, the impact of ZnO NPs exposure on developing embryo. This was surprising, as our initial studies on polarised Caco-2 cell culture, which mimicked functional intestinal epithelium, showed no variation in the gene expression in response to oxide NPs exposure (data unpublished, pending patent application). In a parallel study conducted on a Danio rerio model, where the effects on changes in gene expression were compared following the administration of ZnO NPs and Zn^2+^ in the form of ZnSO_4,_ showed a higher level of change for ZnSO_4_ than NPs [68]. Our results suggest a greater effect on the shape of the nanoparticles than on the Zn^2+^ ions themselves, urging the need for further embryotoxicity studies in regard to nanoparticle safety.

## 5. Conclusions

The comprehensive physicochemical characterisation of ZnO and ZnO with a content of 0.5%mol. Eu NPs synthesised via different hydrothermal routes demonstrated the significant influence of precursor chemistry and rare-earth doping on structural, morphological, and optical properties of obtained NPs. XRD analysis confirmed that pure ZnO crystallises in the hexagonal wurtzite structure (ZnO LONG), which seems to disallow doping with Eu ions. SEM imaging revealed a pronounced morphological transformation from elongated hexagonal prisms in undoped ZnO LONG to oval-like structures in Eu-doped ZnO OVAL nanoparticles, accompanied by a particle size and shape ratio reduction. XRD diffractograms of ZnO OVAL reveal no detectable europium-containing secondary phases. A shift in diffraction peaks toward higher 2θ angles was observed for Eu-doped ZnO OVAL, which suggests lattice compression, possibly due to surface segregation of Eu^3+^ ions, interstitial incorporation, or stress induced by charge compensation defects. These effects are attributed to the combination of europium doping and the use of zinc nitrate as a precursor, which together promote defect formation and alter crystal growth mechanisms. Elemental analysis by EDX confirmed the near-stoichiometric Zn/O ratio in undoped samples and the successful incorporation of europium in doped systems at trace levels. Optical characterisation by CL and PL highlighted the presence of prominent deep-level emissions (DLE), particularly in ZnO OVAL, indicating increased defect densities and efficient non-radiative recombination channels associated with Eu doping. The detection of sharp emission lines assigned to intra-4f transitions of Eu^3+^ ions, notably the hypersensitive ^5^D_0_ → ^7^F_2_ transition, confirmed the successful incorporation of Eu^3+^ into the ZnO host. Spectral analysis further suggests that a significant fraction of Eu^3+^ ions occupy substitutional Zn^2+^ sites with C_3v_ symmetry. At the same time, other broad emission features indicate the presence of europium in multiple non-equivalent environments, including defect-rich or surface-associated sites. Overall, the study underscored the critical role of synthetic parameters and doping in tailoring the properties of ZnO-based nanomaterials. A confocal study showed the predominant fluorescence associated with surface defects of ZnO crystallites following Eu doping in the blue 430–450 nm channel at 405 nm excitation, with just a trace of Eu-specific red fluorescence observed at 543 nm excitation, indistinguishable from the tissue autofluorescence. This allowed direct confirmation of NPs presence in the embryonic tissues for ZnO OVAL.

Embryo exposure to both ZnO OVAL and ZnO LONG nanoparticles induced significant changes in oxidative stress parameters and affected the gene expression levels. The overwhelming impact of ZnO NPs on the genomic level showed how little is known of the possible effects of nanomaterials and how unreliable the studies conducted on the in vitro models are. In the case of ZnO OVAL, the dynamics in both NPs trafficking and response to oxidative stress suggest that their rapid diffusion, transmembrane trafficking and organism circulation may be at the bases of observed oxidative stress and gene expression changes. The most intriguing result was the association of the dynamic Zn removal with the development and functionalisation of kidneys in the chicken embryo, confirmed by confocal imaging for ZnO OVAL, which may indicate different NPs trafficking mechanisms in birds and mammals, or during embryonic and adult life. For ZnO LONG, regardless of higher in vitro toxicity, the observed dynamics of NPs trafficking and their influence on embryonic tissues (both oxidative stress and gene expression levels) were delayed and reduced, suggesting not only the effect of shape, but also possible influence of their anisotropic structure and heterogeneous surface charge distribution on their diffusion and intracellular and intra-organism distribution.

## Figures and Tables

**Table 1:** Selected synthesis parameters of ZnO OVAL (doped with Eu) and ZnO LONG nanoparticles.

Designation of Samples	Shape	Zinc Precursor	Dopant Precursor	Precipitant Agent	Pressure of Synthesis [MPa]	Solution pH
ZnO OVAL	oval	Zinc nitrate Zn(NO_3_)_2_ · 6H_2_O (Sigma-Aldrich, Poznań, Poland)	europium nitrate (V)	ammonia solution (25% Carl Roth, Karlsruhe, Germany)	8	10
ZnO LONG	long	zinc acetate C_4_H_6_O_4_Zn (Roth) (Sigma-Aldrich, Poznań, Poland)	-	ammonia solution (25% Carl Roth, Karlsruhe, Germany)	6	10

**Table 2:** Hydrodynamic size and polydispersity index (PDI) of ZnO nanoparticle suspensions measured at 25 °C.

Sample	Z-Average Diameter (nm) ± SD	PDI
ZnO OVAL	369 ± 7	0.1854
ZnO LONG	2845 ± 95	0.2008

**Table 3:** Changes in gene expression (their individual transcripts) determined by microarrays, which were validated by quantitative real-time PCR. Gene expression changes are expressed as a Fold change (FC) day 4 to day 5 and FDR (corrected) *p* value < 0.01.

Gene Symbol	ZnO OVAL		ZnO LONG		CTRL	
	FC	Regulation	FC	Regulation	FC	Regulation
*COL6A2*	4.19	down			3.43	down
*COL6A2*					4.27	down
*COL6A2*					4.42	down
*STEAP3*	3.28	up	3.37	up		
*STEAP3*	2.88	up				
*PSMB7*	2.56	up	3.10	up		

## Data Availability

The microarray data presented in this study are openly available in the Gene Expression Omnibus data repository under the accession number: GSE301197. Original, unprocessed confocal microphotographs are attached as the Supplementary Materials to this article.

## Supplementary Materials

The following supporting information can be downloaded at: https://www.mdpi.com/article/10.3390/nano15181412/s1, Figure S1: Confocal microscopy image of chicken embryo tissues from head region following administration of ZnO OVAL NPs at concentration 10 µg/mL at day 5 of incubation. Lens magnification 20×. Red fluorescence corresponds to nuclei stained with 7-AAD, while blue fluorescence indicates ZnO OVAL NPs; Figure S2: Confocal microscopy image of chicken embryo tissues from mid body region following administration of ZnO OVAL NPs at concentration 10 µg/mL at day 5 of incubation. Lens magnification 20×. Red fluorescence corresponds to nuclei stained with 7-AAD, while blue fluorescence indicates ZnO OVAL NPs; Figure S3: Confocal microscopy image of chicken embryo tissues following administration of ZnO OVAL NPs at concentration 10 µg/mL at day 7 of incubation. Lens magnification 20×. Red fluorescence corresponds to nuclei stained with 7-AAD, while blue fluorescence indicates ZnO OVAL NPs; Figure S4: Confocal microscopy image of chicken embryo tissues following administration of ZnO OVAL NPs at concentration 10 µg/mL at day 7 of incubation. Lens magnification 20×. Red fluorescence corresponds to nuclei stained with 7-AAD, while blue fluorescence indicates ZnO OVAL NPs; Figure S5: Confocal microscopy image of chicken embryo tissues following administration of ZnO OVAL NPs at concentration 10 µg/mL at day 10 of incubation. Lens magnification 20×. Red fluorescence corresponds to nuclei stained with 7-AAD, while blue fluorescence indicates ZnO OVAL NPs; Figure S6: Confocal microscopy image of chicken embryo tissues following administration of ZnO OVAL NPs at concentration 10 µg/mL at day 10 of incubation. Lens magnification 20×. Red fluorescence corresponds to nuclei stained with 7-AAD, while blue fluorescence indicates ZnO OVAL NPs; Figure S7: Confocal microscopy image of chicken embryo tissues from head region following administration of ZnO OVAL NPs at concentration 100 µg/mL at day 5 of incubation. Lens magnification 20×. Red fluorescence corresponds to nuclei stained with 7-AAD, while blue fluorescence indicates ZnO OVAL NPs; Figure S8: Confocal microscopy image of chicken embryo tissues from mid body region following administration of ZnO OVAL NPs at concentration 100 µg/mL at day 5 of incubation. Lens magnification 20×. Red fluorescence corresponds to nuclei stained with 7-AAD, while blue fluorescence indicates ZnO OVAL NPs; Figure S9: Confocal microscopy image of chicken embryo tissues following administration of ZnO OVAL NPs at concentration 100 µg/mL at day 7 of incubation. Lens magnification 20×. Red fluorescence corresponds to nuclei stained with 7-AAD, while blue fluorescence indicates ZnO OVAL NPs; Figure S10: Confocal microscopy image of chicken embryo tissues following administration of ZnO OVAL NPs at concentration 100 µg/mL at day 7 of incubation. Lens magnification 20×. Red fluorescence corresponds to nuclei stained with 7-AAD, while blue fluorescence indicates ZnO OVAL NPs; Figure S11: Confocal microscopy image of chicken embryo tissues following administration of ZnO OVAL NPs at concentration 100 µg/mL at day 10 of incubation. Lens magnification 20×. Red fluorescence corresponds to nuclei stained with 7-AAD, while blue fluorescence indicates ZnO OVAL NPs; Figure S12: Confocal microscopy image of chicken embryo tissues following administration of ZnO OVAL NPs at concentration 100 µg/mL at day 10 of incubation. Lens magnification 20×. Red fluorescence corresponds to nuclei stained with 7-AAD, while blue fluorescence indicates ZnO OVAL NPs.

## Abbreviations

The following abbreviations are used in this manuscript:

7-AAD	7-aminoactinomycin D
AAS	atomic absorption spectrometry
BHT	butylated hydroxytoluene
CD50	cell death 50%
CEFs	(primary) chicken embryo fibroblasts
CL	cathodoluminescence
CNCCs	cranial neural crest cells
CP	carbonylated proteins
CTRL	control
DLE	deep level emission
DLS	dynamic light scattering
DMEM	Dulbecco’s modified eagle medium
DNPH	2,4-dinitrophenylhydrazine
DTT	ditiotreitol
EDTA	ethylene-diaminetetraacetic acid
ELS	electrophoretic light scattering
EDX	energy dispersive X-ray
FBS	foetal bovine serum
MCS	mean crystallite sizes
MDA	malondialdehyde
NBE	near band edge
NBT	nitro blue tetrazolium chloride
NPs	nanoparticles
PBS	phosphate-buffered saline
PDI	polydispersity indices
PL	photoluminescence emission
PLE	photoluminescence excitation
PMSF	phenylmethanesulfonyl fluoride
PTMs	post-translational modifications
ROS	reactive oxygen species
RT	room temperature
SEM	scanning electron microscopy
SOD	superoxide dismutase
TBA	thiobarbituric acid
TCA	trichloroacetic acid
XRD	X-ray Diffraction
