# Supplementary material for: Zinc Oxide Nanoparticles Affect the Genomic and Redox Status of Chicken Embryo—Influence of Shape
Source: Nanomaterials (Basel). 2025 Sep 13;15(18):1412. doi: 10.3390/nano15181412 (PMC12472910; doi:10.3390/nano15181412)
Supplement: Supplementary file 1 [file nanomaterials-15-01412-s001.zip › nanomaterials-3852285-supplementary.pdf]

Supplementary information:

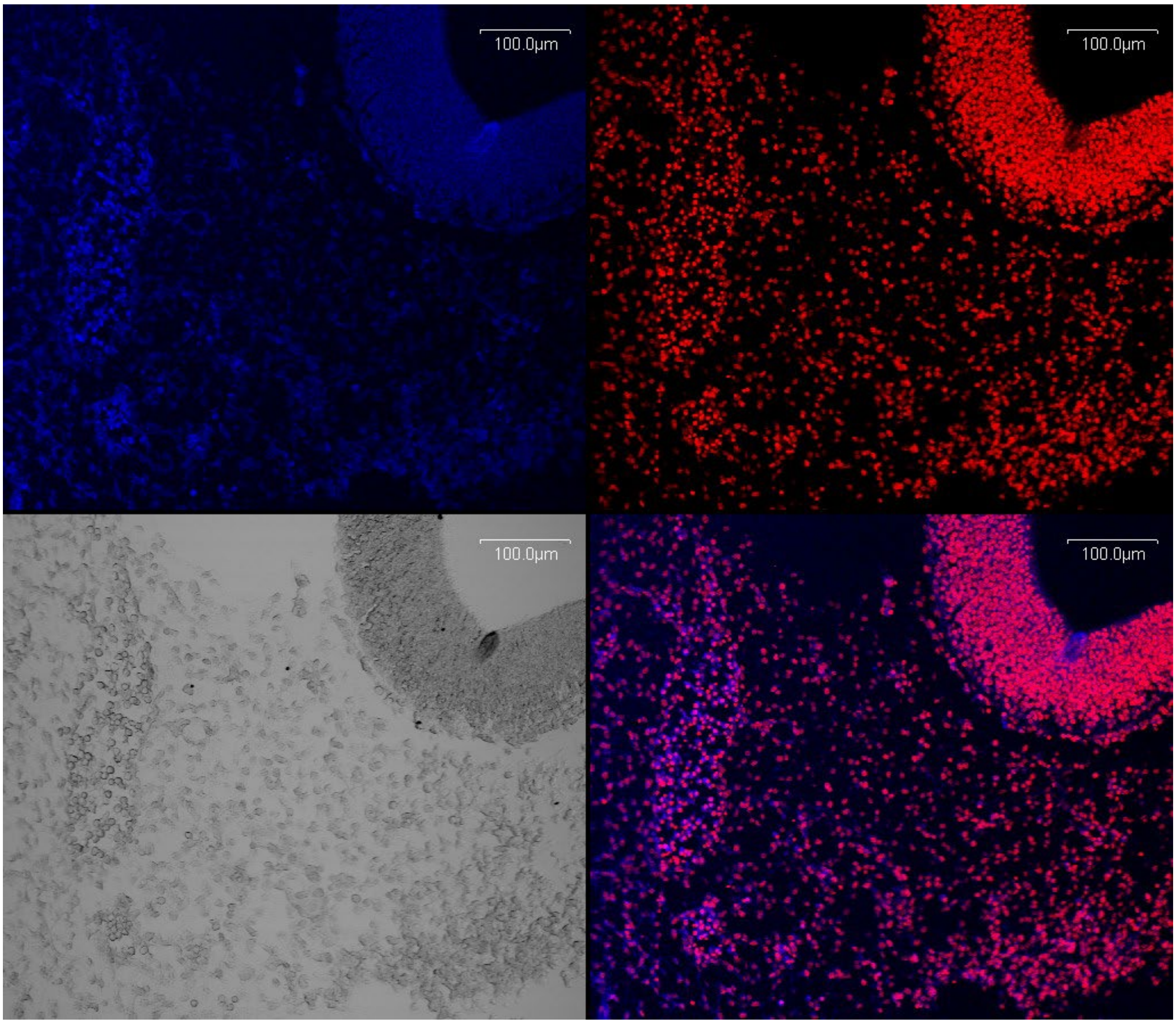

Figure S1. Confocal microscopy image of chicken embryo tissues from head region following administration of ZnO OVAL NPs at concentration 10 µg/ml at day 5 of incubation. Lens magnification 20x. Red fluorescence corresponds to nuclei stained with 7-AAD, while blue fluorescence indicates ZnO OVAL NPs.

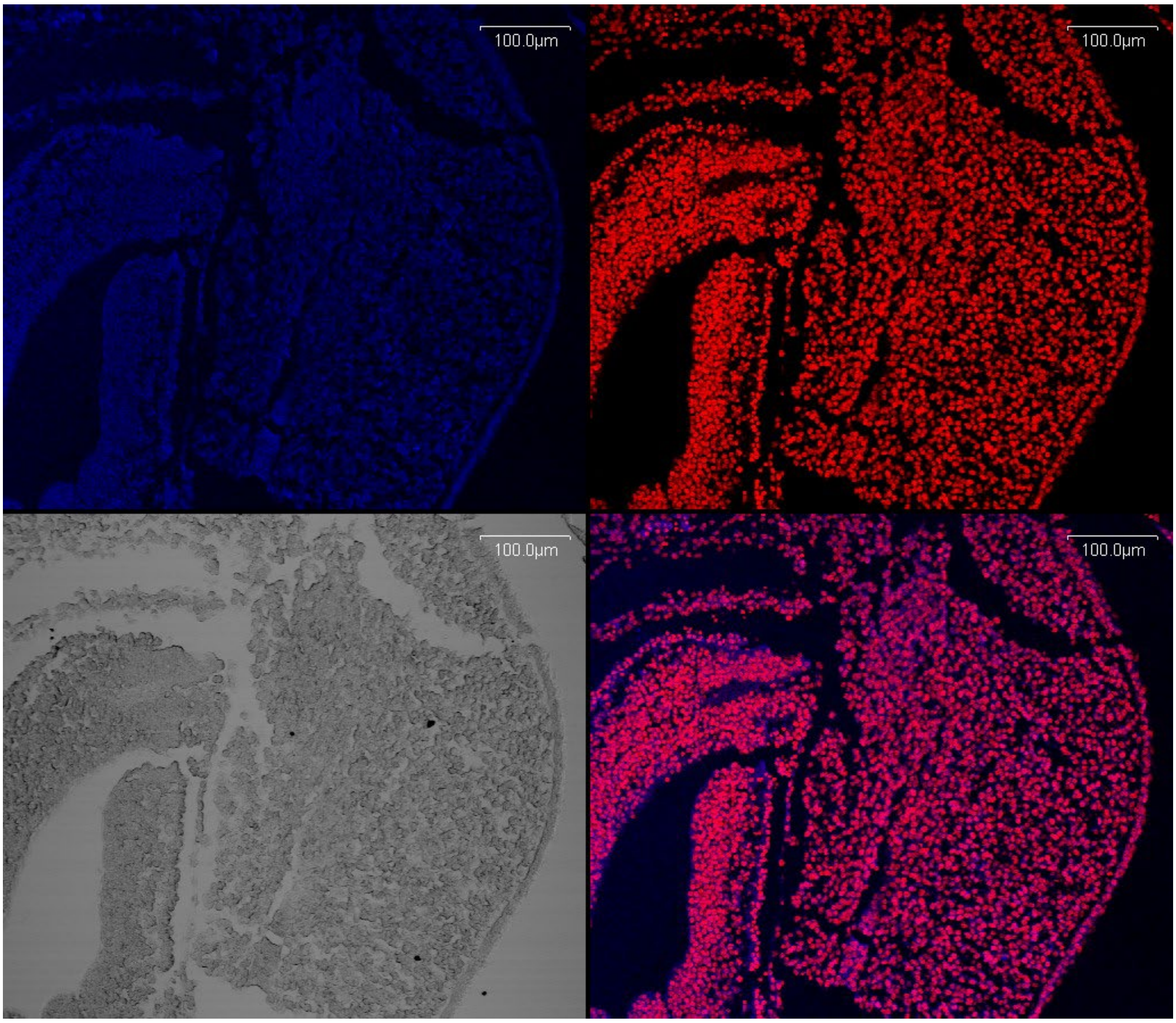

Figure S2. Confocal microscopy image of chicken embryo tissues from mid body region following administration of ZnO OVAL NPs at concentration 10 μg/ml at day 5 of incubation. Lens magnification 20x. Red fluorescence corresponds to nuclei stained with 7-AAD, while blue fluorescence indicates ZnO OVAL NPs.

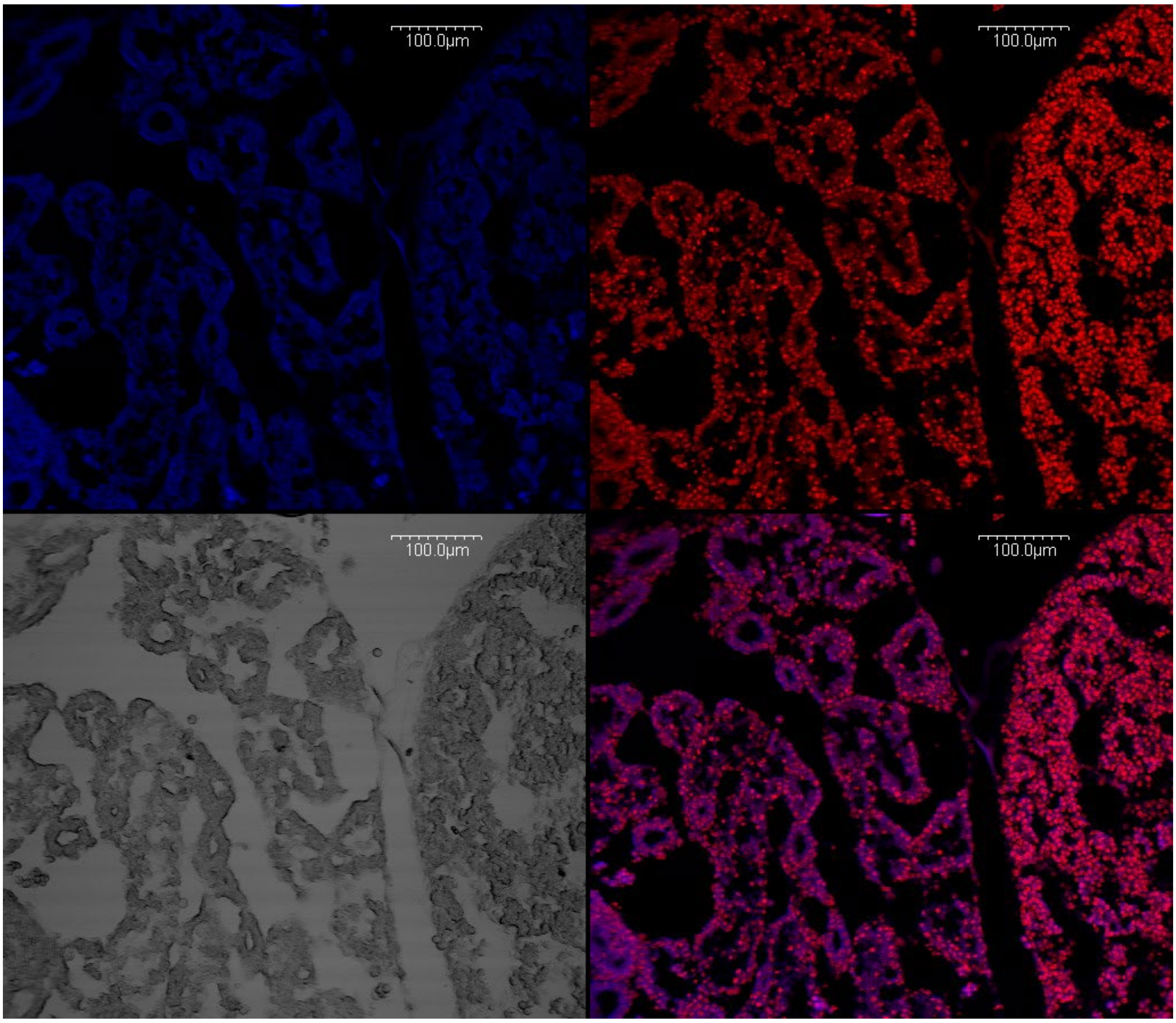

Figure S3. Confocal microscopy image of chicken embryo tissues following administration of ZnO OVAL NPs at concentration 10 µg/ml at day 7 of incubation. Lens magnification 20x. Red fluorescence corresponds to nuclei stained with 7-AAD, while blue fluorescence indicates ZnO OVAL NPs.

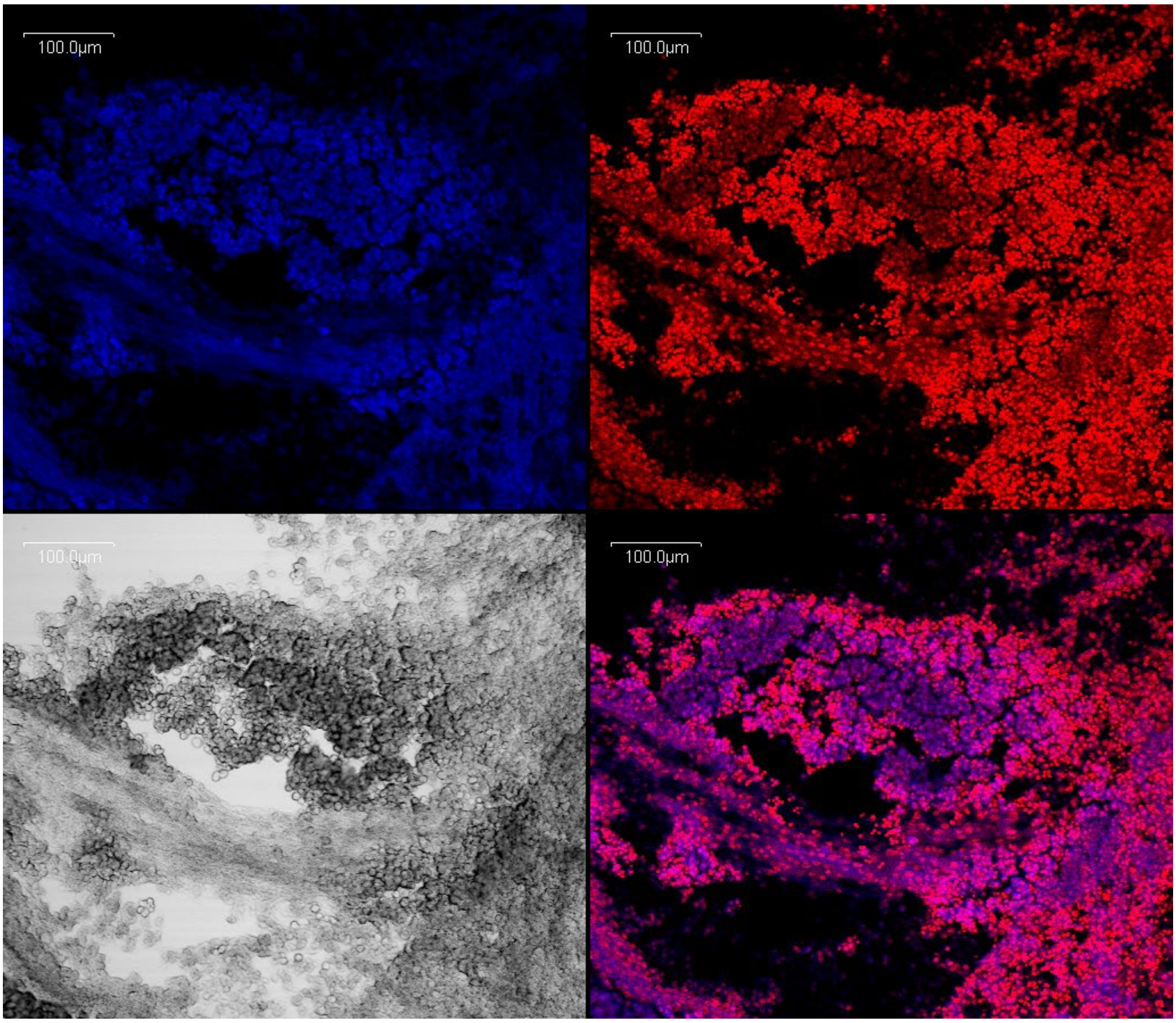

Figure S4. Confocal microscopy image of chicken embryo tissues following administration of ZnO OVAL NPs at concentration 10 µg/ml at day 7 of incubation. Lens magnification 20x. Red fluorescence corresponds to nuclei stained with 7-AAD, while blue fluorescence indicates ZnO OVAL NPs.

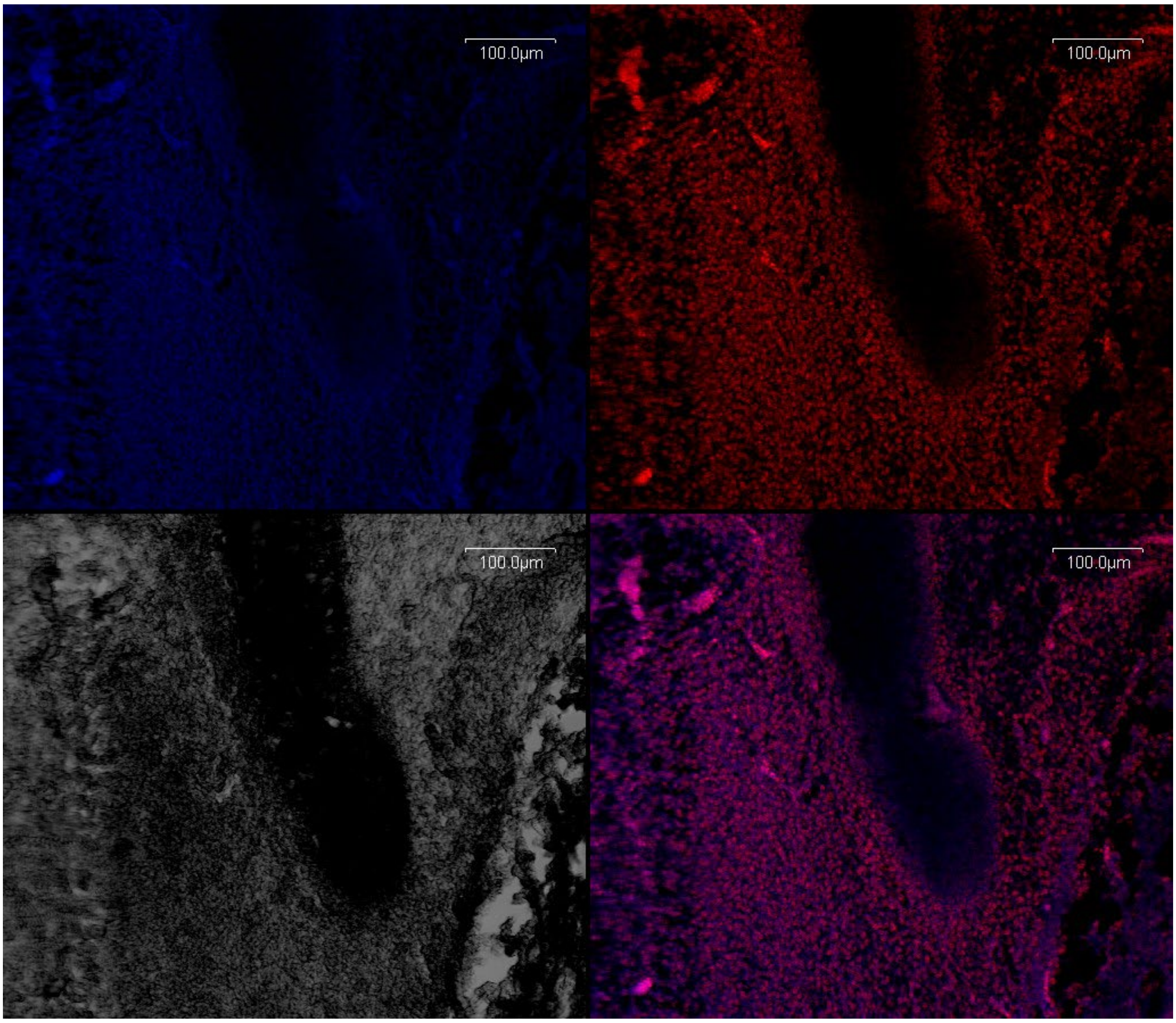

Figure S5. Confocal microscopy image of chicken embryo tissues following administration of ZnO OVAL NPs at concentration 10 µg/ml at day 10 of incubation. Lens magnification 20x. Red fluorescence corresponds to nuclei stained with 7-AAD, while blue fluorescence indicates ZnO OVAL NPs.

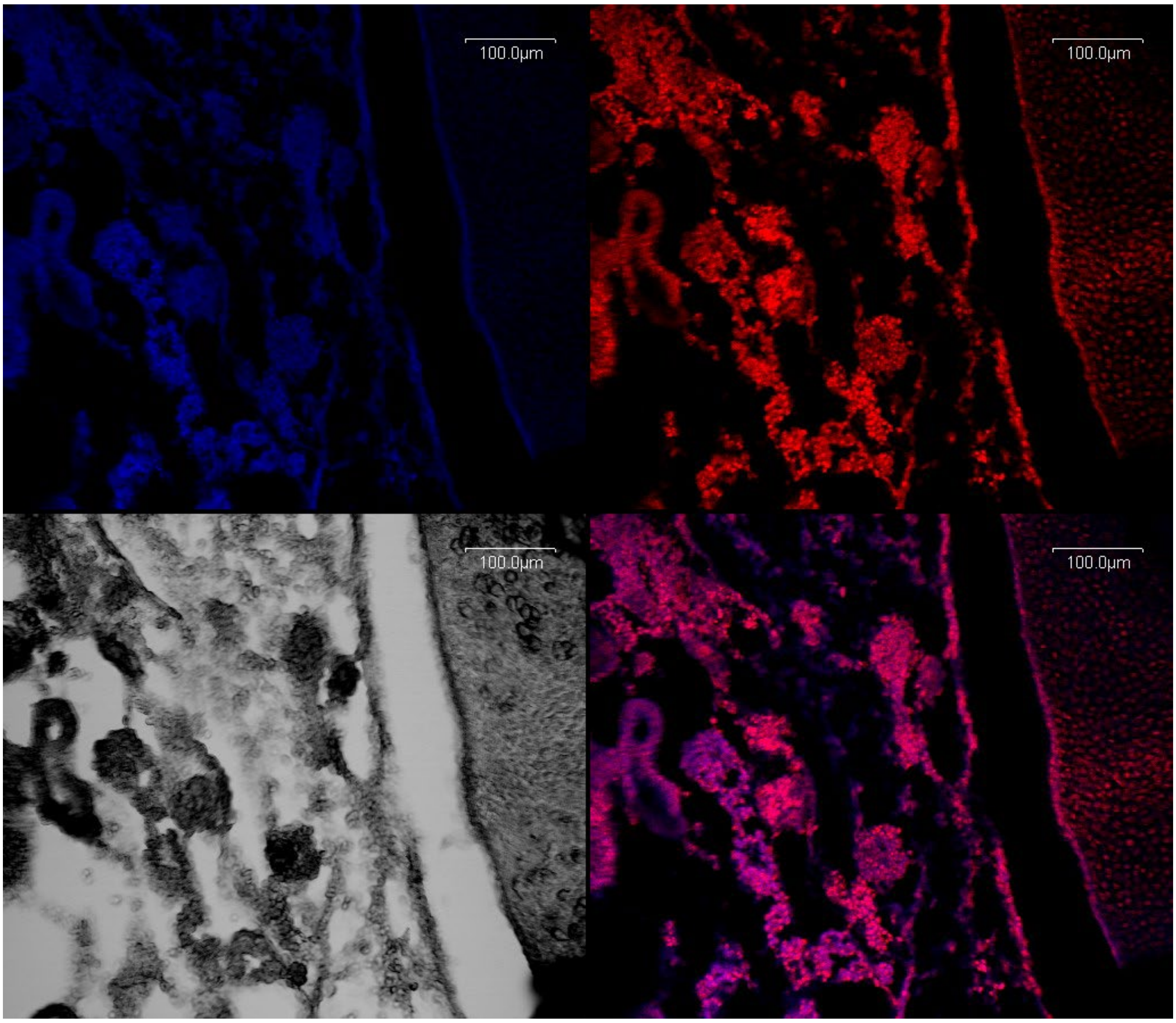

Figure S6. Confocal microscopy image of chicken embryo tissues following administration of ZnO OVAL NPs at concentration 10 μg/ml at day 10 of incubation. Lens magnification 20x. Red fluorescence corresponds to nuclei stained with 7-AAD, while blue fluorescence indicates ZnO OVAL NPs.

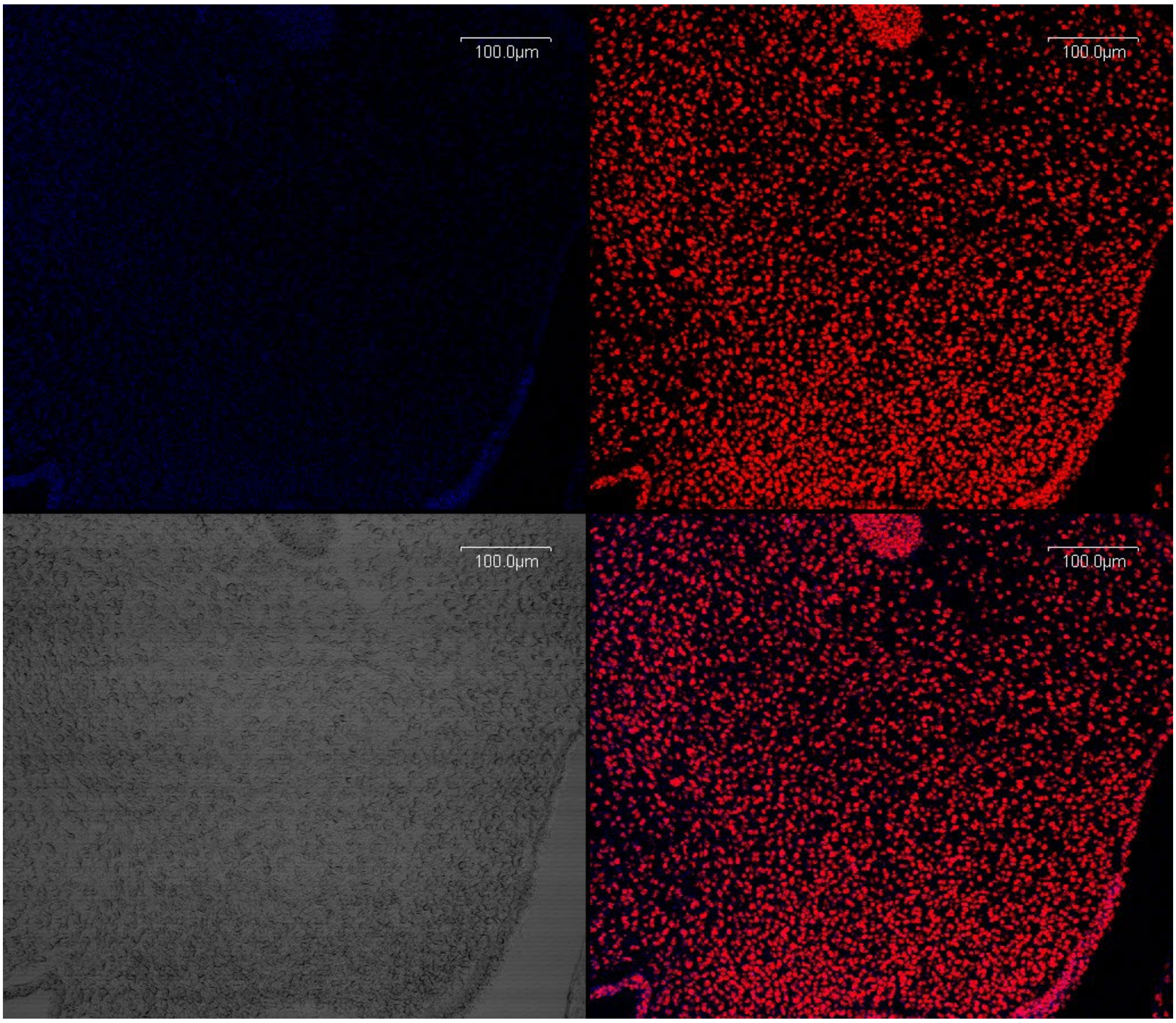

Figure S7. Confocal microscopy image of chicken embryo tissues from head region following administration of ZnO OVAL NPs at concentration 100 µg/ml at day 5 of incubation. Lens magnification 20x. Red fluorescence corresponds to nuclei stained with 7-AAD, while blue fluorescence indicates ZnO OVAL NPs.

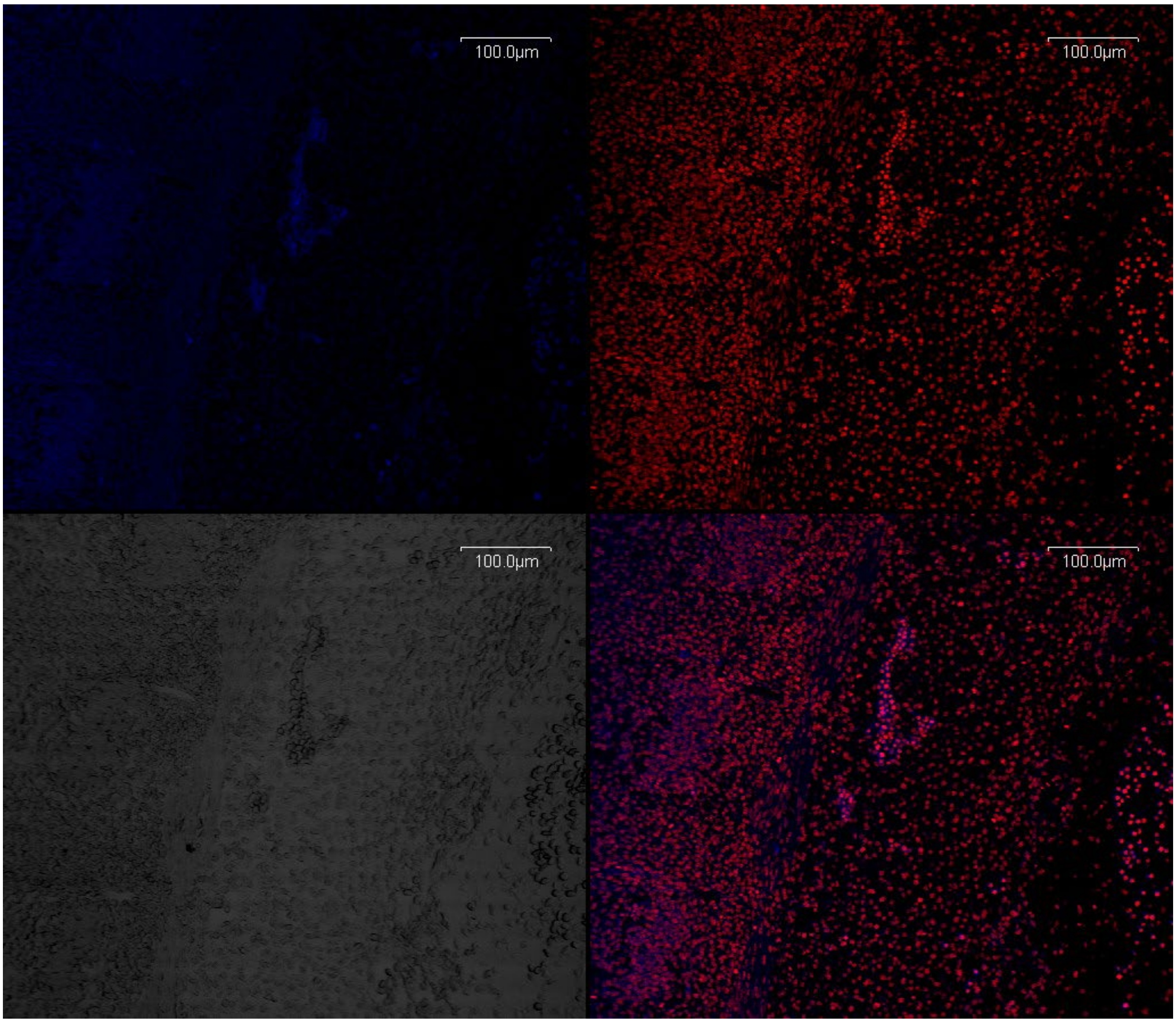

Figure S8. Confocal microscopy image of chicken embryo tissues from mid body region following administration of ZnO OVAL NPs at concentration 100 µg/ml at day 5 of incubation. Lens magnification 20x. Red fluorescence corresponds to nuclei stained with 7-AAD, while blue fluorescence indicates ZnO OVAL NPs.

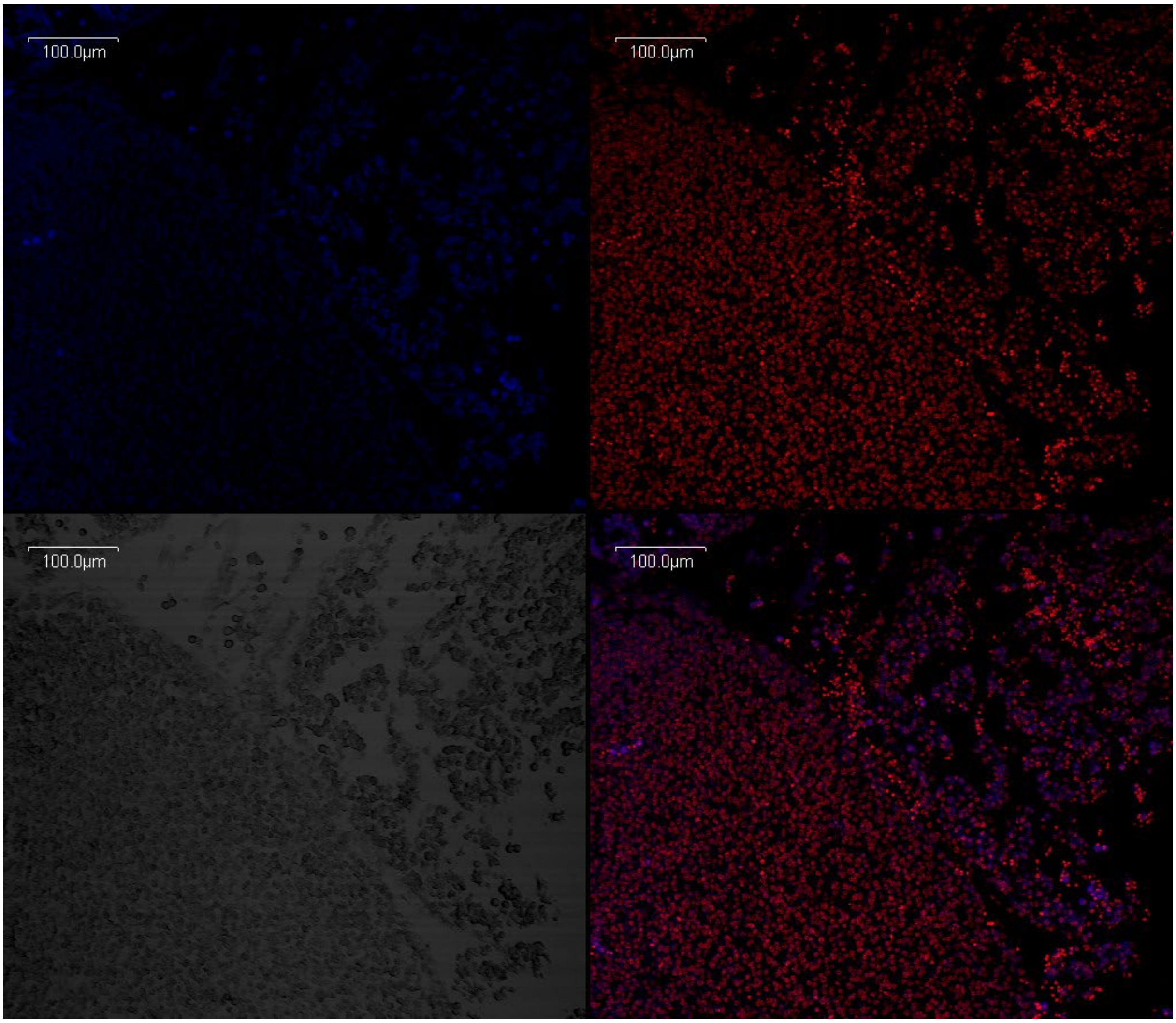

Figure S9. Confocal microscopy image of chicken embryo tissues following administration of ZnO OVAL NPs at concentration 100 μg/ml at day 7 of incubation. Lens magnification 20x. Red fluorescence corresponds to nuclei stained with 7-AAD, while blue fluorescence indicates ZnO OVAL NPs.

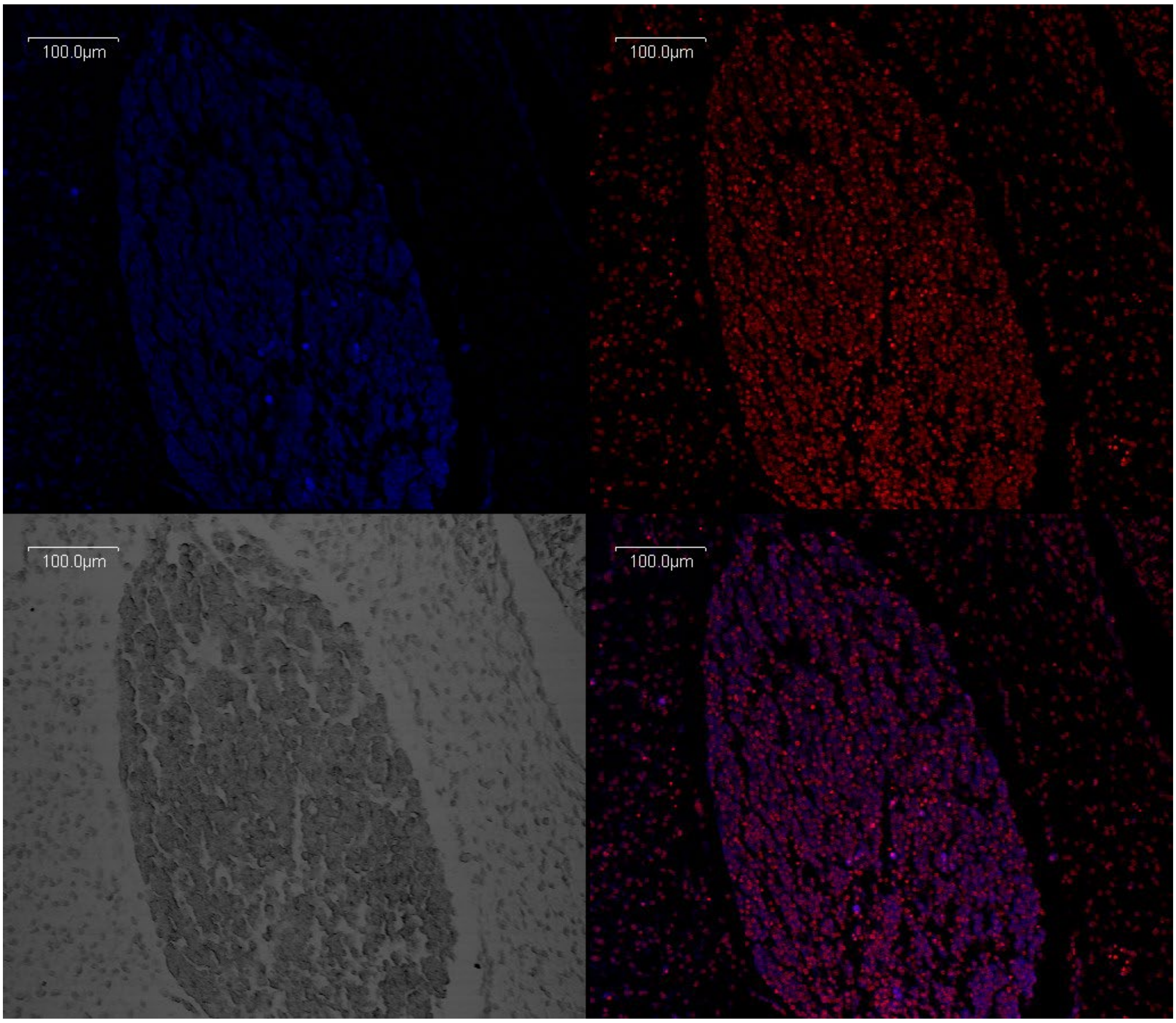

Figure S10. Confocal microscopy image of chicken embryo tissues following administration of ZnO OVAL NPs at concentration 100 μg/ml at day 7 of incubation. Lens magnification 20x. Red fluorescence corresponds to nuclei stained with 7-AAD, while blue fluorescence indicates ZnO OVAL NPs.

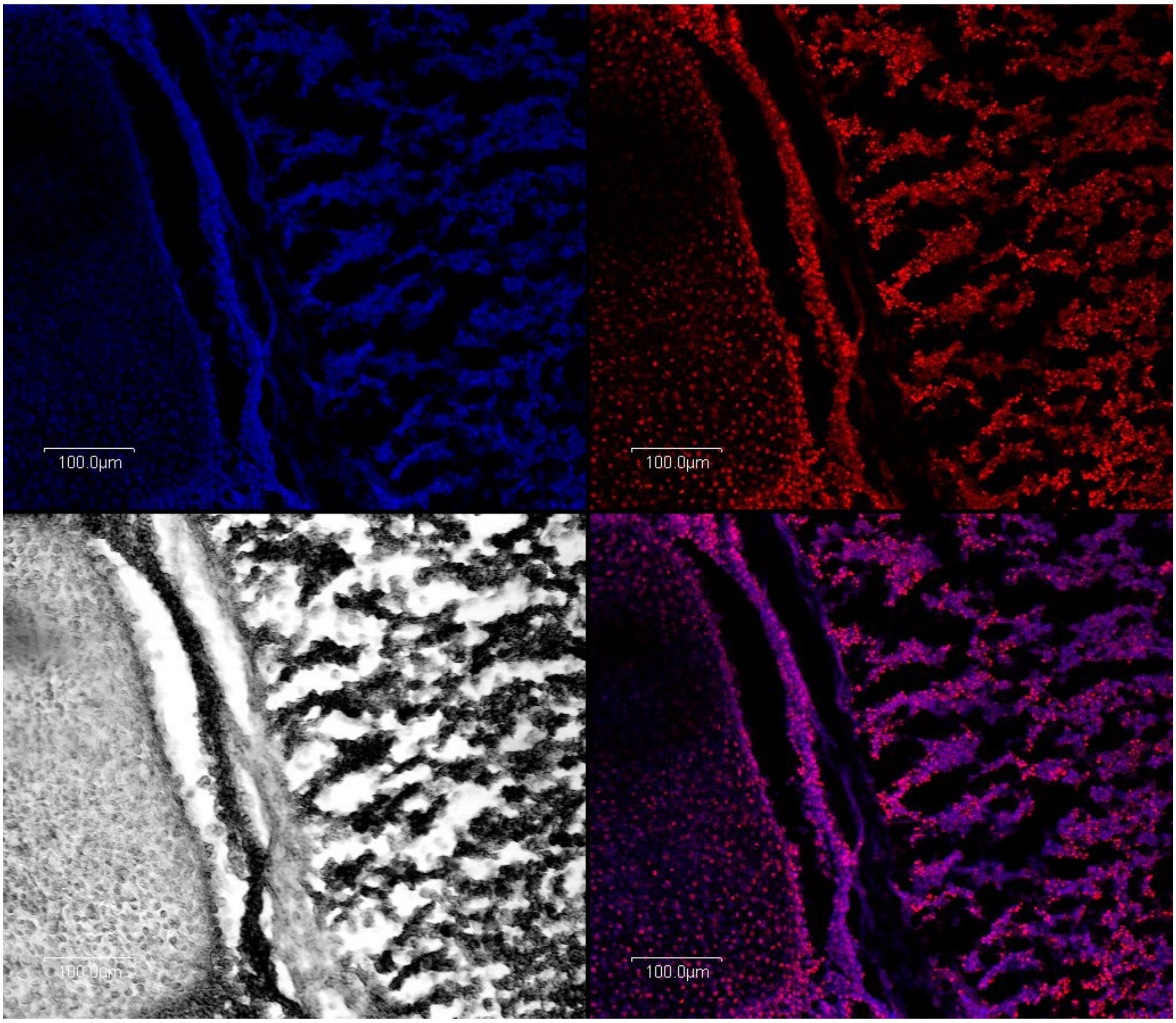

Figure S11. Confocal microscopy image of chicken embryo tissues following administration of ZnO OVAL NPs at concentration 100 µg/ml at day 10 of incubation. Lens magnification 20x. Red fluorescence corresponds to nuclei stained with 7-AAD, while blue fluorescence indicates ZnO OVAL NPs.

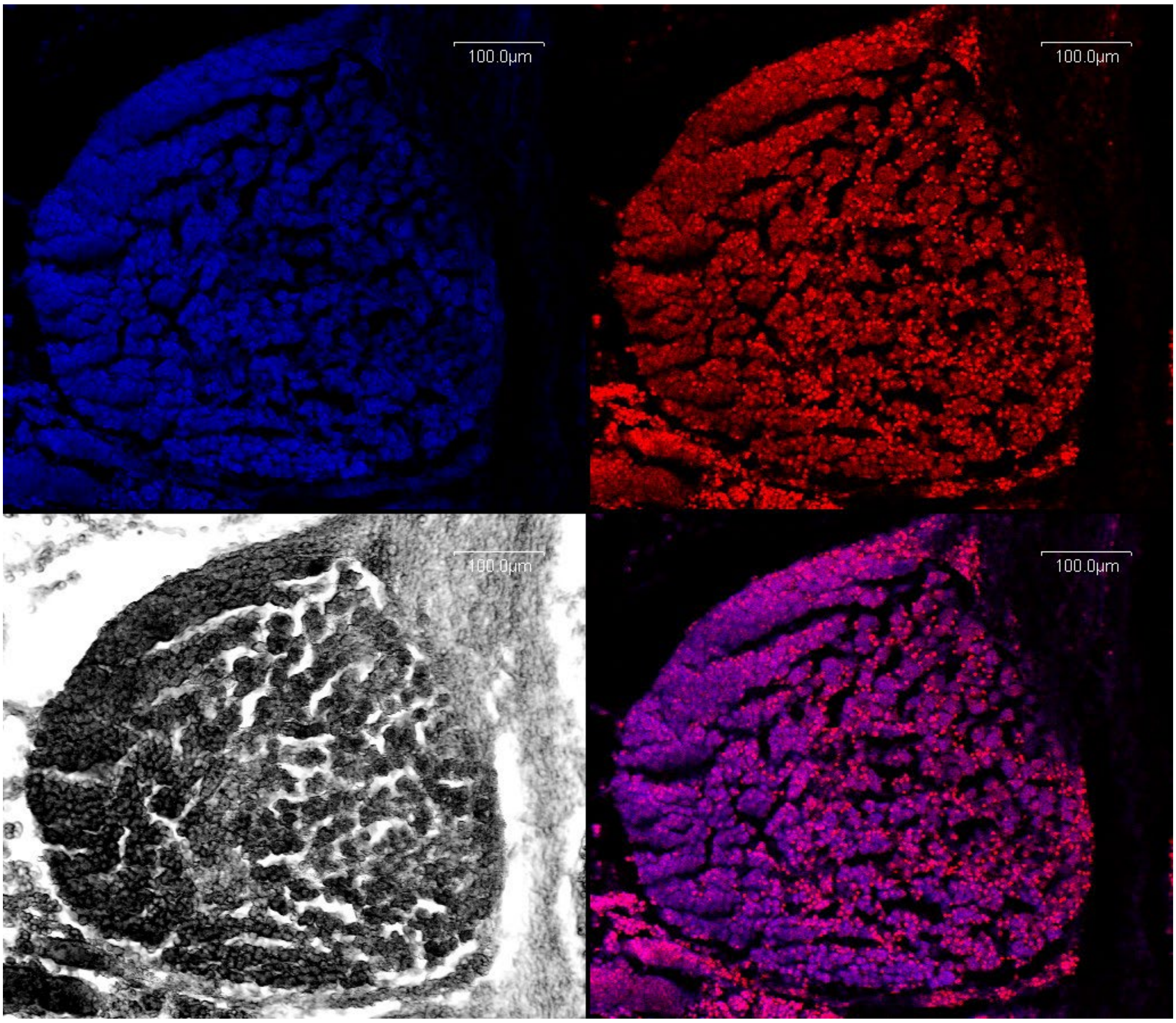

Figure S12. Confocal microscopy image of chicken embryo tissues following administration of ZnO OVAL NPs at concentration 100 μg/ml at day 10 of incubation. Lens magnification 20x. Red fluorescence corresponds to nuclei stained with 7-AAD, while blue fluorescence indicates ZnO OVAL NPs.
